# Thanatin and vinyl sulfide analogues as narrow spectrum antimicrobial peptides that synergise with polymyxin B

**DOI:** 10.3389/fphar.2024.1487338

**Published:** 2024-11-05

**Authors:** Oscar A. Shepperson, Paul W. R. Harris, Margaret A. Brimble, Alan J. Cameron

**Affiliations:** ^1^ School of Chemical Sciences, The University of Auckland, Auckland, New Zealand; ^2^ School of Biological Sciences, The University of Auckland, Auckland, New Zealand; ^3^ Maurice Wilkins Centre for Molecular Bio-Discovery, The University of Auckland, Auckland, New Zealand

**Keywords:** β-hairpin, thanatin, polymyxin B, antibiotic synergy, vinyl sulfide, AMP

## Abstract

Thanatin is a β-hairpin antimicrobial peptide cyclised by a single disulfide bond that has shown potent broad-spectrum activity towards bacterial and fungal pathogens. Towards Gram-negative species, thanatin acts both by forming trans-membranal pores and inhibiting outer membrane biogenesis by binding to LptA and blocking lipopolysaccharide (LPS) transport. Inspired by previous modifications of thanatin, an analogue was prepared which demonstrated potent but selective activity towards *E. coli*. Furthermore, this compound was shown to act in synergy with the highly potent FDA-approved lipopeptide antibiotic polymyxin B, which engages LPS at the cytoplasmic membrane. Four analogues of thanatin in which the disulfide was substituted for vinyl sulfide bridge mimetics were prepared, all of which retained similar secondary structures. Two of these retained substantial potency and selectivity towards *E. coli*. Importantly, synergy with polymyxin B was also maintained for the lead analogue. The vinyl sulfide potentially offers a facile replacement strategy for labile disulfide bonds and the selective activity and drug synergy of the reported thanatin analogues is promising for the development of narrow spectrum antimicrobials with reduced likelihood of resistance emerging in clinical settings.

## Introduction

Antimicrobial resistance (AMR) represents a significant global health threat, characterised by the ability of microorganisms (e.g. bacteria, viruses, fungi, and parasites) to withstand the effects of medications traditionally used to eradicate them or inhibit their proliferation (Centers for Disease Control and Prevention, 2019; Murray et al., 2022). This resistance compromises the efficacy of standard treatments, leading to prolonged infections, increased medical expenses, and heightened mortality rates. The escalation of AMR is fuelled by factors including the overuse and misuse of antibiotics, insufficient infection control measures, and a stagnation in the development of novel antibiotics. Antimicrobial peptides (AMPs) have gained increasing attention as potential alternatives or adjuncts to conventional antibiotics in addressing AMR (Malik and Bhattacharyya, 2019).

AMPs are short (<50 AAs), naturally occurring peptides, that form a crucial part of the innate immune system across a wide range of organisms (Hancock et al., 2016). These peptides most commonly exhibit broad-spectrum antimicrobial activity, primarily through mechanisms that involve the disruption of microbial membranes, leading to cell lysis and death (Fjell et al., 2012; Mahlapuu et al., 2016). This membrane-targeting action is less prone to inducing resistance when compared to traditional antibiotics that often target proteins with specific cellular functions (Li et al., 2018; Yu et al., 2020). Furthermore, some classes of AMPs (e.g. β-hairpins) have been shown to exhibit multimodal mechanisms of action, alongside cell lysis, further rendering them less susceptible to resistance development (Krieger et al., 2022).

To enhance therapeutic outcomes and mitigate the risk of resistance, the use of combination antimicrobial therapies is being increasingly explored (Mhlongo et al., 2023). This strategy typically involves the use of two or more therapeutic agents, which can act synergistically to increase antimicrobial efficacy, reduce required dosages, and potentially lower toxicity. There are two primary approaches for combining different treatments; 1) combination therapies, 2) covalent conjugates.

**FIGURE 1:**
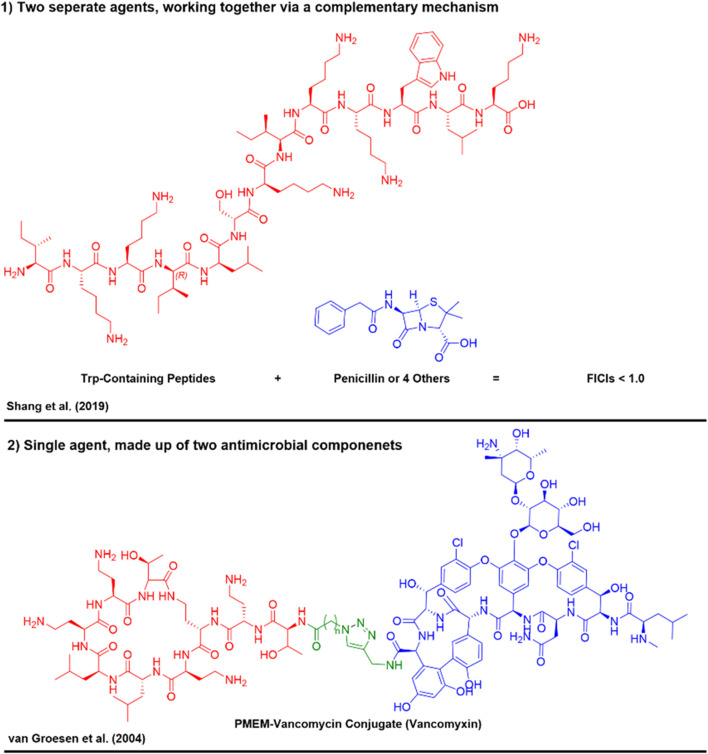
Example of the two types of combination therapies aimed to target antimicrobials and overcome resistance mechanisms (Shang et al., 2019; van Groesen et al., 2021).

1) The administration of two separate antimicrobial agents, that ultimately work together, via complementary mechanisms, resulting in the concentrations/dose co-administered being below that of which they would normally be required to elicit the same antimicrobial effect (Figure 1) (Kalan and Wright, 2011). A great example of this is the co-administration of Trp rich membrane disrupting peptides alongside commonly used antibiotics (e.g. Penicillin’s) (Shang et al., 2019). Through membrane lysis by the Trp rich peptides, the conventional antibiotics can more effectively reach their target, boosting their activity.
2) The administration of a single antimicrobial agent, composed of two agents/chemical portions of agents covalently bound to one another. Each portion of the conjugate acts cooperatively to elicit a greater antimicrobial effect than either of the agents do alone. An example of this approach is the covalent bonding of AMPs to form hybrid molecules (van Groesen et al., 2021; Wood et al., 2021). These covalently bound combination AMPs can integrate the properties of individual peptides, potentially enhancing potency, stability, and specificity in targeting resistant pathogens. By leveraging such molecular combinations, researchers have aimed to develop innovative treatments that offer robust efficacy against a broad spectrum of resistant organisms, while also curtailing the emergence of resistance (Figure 1) (Luther et al., 2019). Examples of such strategies include not only chimeric or hybrid AMPs, but also cell-penetrating peptide (CPP) conjugates with AMPs (Lee et al., 2019), small molecules antibiotics (Hadjicharalambous et al., 2022) and peptide nucleic acids (PNAs) (Yamamoto et al., 2022). Gene silencing with CPP-PNA conjugates has recently been shown as a strategy to enhance permeability of antibiotics to Gram-negative species (Yamamoto et al., 2022).

**FIGURE 2:**
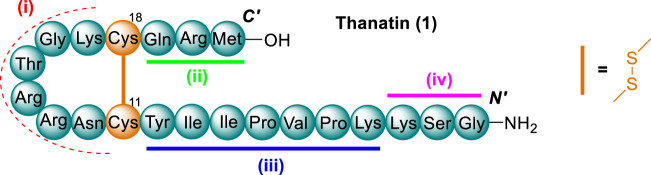
AMP thanatin (1), represented by three letter code, disulfide bond between residues Cys^11^ and Cys^18^ shown in orange. Regions essential for antimicrobial activity highlighted as follows; i) *C-*terminal loop (red), ii) the *C*-terminal three residue extension (green), iii) a stretch of seven *N-*terminal mostly hydrophobic residues (blue) and iv) the three final *N-*terminal residues (pink).

Thanatin was initially discovered following the immune challenge of the insect, *Podisus maculiventris* (spined soldier bug) (Fehlbaum et al., 1996). Thanatin (**1**) is comprised of 21-residues and maintains a β-hairpin secondary structure via an intramolecular disulfide bond between residues Cys^11^ and Cys^18^ (Figure 2). It showed a broad spectrum of activity towards Gram-negative and Gram-positive bacteria as well as fungi (Fehlbaum et al., 1996). Interestingly, thanatin exhibited no antimicrobial activity towards *S. aureus,* despite showing activity against a variety of other Gram-positive pathogens (e.g., *A. viridians* and *M. luteus*). A few of these key aspects identified during initial structure-activity relationship (SAR) studies of thanatin were, the determination that *C-*terminal amidation enhanced the spectrum of activity, the d enantiomer was dramatically less active toward gram negative pathogens, and that four regions of thanatin are required for full antimicrobial potency. These four regions identified were as follows; the *C-*terminal loop, the *C-*terminal three-residue extension, a stretch of seven *N*-terminal mostly hydrophobic residues. Notably, the three final *N*-terminal residues are required for antifungal but not antibacterial activity (Fehlbaum et al., 1996).

In Gram-negative bacteria, the antimicrobial activity of thanatin (**1**) results from a dual mechanism. In addition to forming trans-membranal pores leading to cell lysis, thanatin (**1**) acts as an inhibitor of the lipopolysaccharide transport (Lpt) system, binding to LptA (Vetterli et al., 2018). During membrane assembly in Gram-negative bacteria, liposaccharides (LPS) are transported to the outer membrane by a series of seven lipopolysaccharide transporter proteins (LptA-G). Inhibition of any of these transporters leads to an inability for the bacteria to transport LPS, inhibiting bacterial outer membrane biogenesis (Okuda et al., 2012; Okuda et al., 2016). LptA was found to form head to tail dimers resulting in protein bridges across the periplasm during LPS transport (Vetterli et al., 2018). Upon superimposing the solution structure of a thanatin-LptA complex with the LptA dimer, overlapping thanatin (**1**) binding was observed (Vetterli et al., 2018). Thanatin is also evidenced to interrupt the LptC-LptA protein-protein interaction (Moura et al., 2020). Accordingly, thanatin (**1**) is believed to prevent the crucial periplasmic bridges from forming, inhibiting LPS transport (Vetterli et al., 2018). This mechanism is consistent with a loss in potency towards Gram-negative pathogens observed by Fehlbaum et al. (1996) when examining the antimicrobial activity of an entirely d-enantiomeric thanatin analogue (Fehlbaum et al., 1996). It has been observed on multiple occasions that for AMPs which block protein-protein interactions, the enantiomeric peptide (l-to d-switching of amino acids) results in significant losses in antimicrobial activity. This results from the inability of the enantiomeric peptide to interact with the chiral target protein, which in the case of thanatin is evidenced to be the LptA transporter (Henriques et al., 2019).

Whilst bacteria are able to modulate their LPS biosynthesis to maintain an abundance in the cell membrane, they do not possess a mechanism to remove LPS. For this reason, LPS targeting antibiotics, such as the polymyxins, remain promising antibacterial agents (Sabnis et al., 2021). Polymyxin B is known to exert its antimicrobial activity after binding to LPS of Gram-negative bacteria in the outer- and subsequently inner-membrane. Unfortunately, despite its approval for clinical use, its utility remains hamstrung by significant toxicity concerns (Liu et al., 2021), limiting clinical use to a last-line of defence that often caused severe nephrotoxic injury in life-saving circumstances. Sabnis et al. (2021) recently demonstrated murepavadin, a head-to-tail cyclised β-hairpin AMP and LptD inhibitor active selectively towards *Pseudomonas aeruginosa* (Dale et al., 2018), caused accumulation of LPS in the cytoplasmic membrane. This resulted in increased sensitivity to polymyxins and enhanced efficacy *in vivo*.

Although polymyxin is currently used as a last line of defence antibiotic it has serious concerns regarding it nephrotoxicity (Na et al., 2024). It has been suggested that by implementing combination therapies that include polymyxin, the effective dose may be minimised, ultimately reducing toxicity. Furthermore, with a number of researchers working towards the development of new less-toxic polymyxins (Aslan et al., 2022), including our own research groups (Harris et al., 2022; Na et al., 2024), these new derivatives may see further benefit from use in synergistic combination therapies, reducing dose requirements and/or the development of resistance. Most noteworthy of these is Spero Therapeutics compound SPR206 (Bruss et al., 2023), which has recently received FDA Fast Track designation and has Phase II clinical trials planned.

Inspired by Sabnis et al. (2021) observing synergy between polymyxin antibiotics and LPS transport inhibitor murepavadin in *P. aeruginosa*, we set out to investigate whether thanatin analogues would also act synergistically with polymyxin B due to the documented inhibitory interaction with LptA in additional species such as *E. coli*. Furthermore, we wanted to investigate whether the disulfide of thanatin (**1**) could be replaced with vinyl sulfides as bridge mimetics (Cameron et al., 2020; Shepperson et al., 2024) and maintain a similar antimicrobial function and more importantly, our hypothesised synergism.

## Materials and methods

### General information

For general procedures and materials see SI. Common chemical abbreviations used throughout include; dimethylformamide (DMF), dichloromethane (CH_2_Cl_2_), amino acid (AA), approximately (approx.), 1,4-dioxane (dioxane), diethyl ether (Et_2_O), 3-butynoic acid (3-BA), solid-phase peptide synthesis (SPPS), 2,4,6-trimethylpyridine (*sym*-collidine), trifluoroacetic acid (TFA), Milli-Q water (MQ H_2_O), *N,N′*-diisopropylethylamine (DIPEA), (7-azabenzotriazol-1-yloxy) trispyrrolidinophosphonium (PyAOP), diemthylsulfoxide (DMSO), triisopropylsilane (TIPS), 1-[bis(dimethylamino) methylene]-1*H*-1,2,3-triazole [4,5-b]pyridinium 3-oxide hexafluorophosphate (HATU), *tert*-butyloxycabonyl (Boc), *tert*-butyl (*t*Bu), acetonitrile (MeCN), fluorenyl-methoxycarbonyl protecting group (Fmoc), pentamethyl-2,3-dihydrobenzofuran-5-sulfonyl (Pbf), triphenylmethyl (Trt), phenylsilane (PhSiH_3_), *N*-ethoxycarbonyl-2-ethoxy-1,2-dihydroquinoline (EEDQ), di-*tert*-butyl decarbonate (Boc_2_O), trifluoroethanol (TFE), phosphate buffered saline (PBS), 5,5-dithio-bis-(2-nitrobenzoic acid) (DTNB, Ellman’s Reagents), 4-[(R,S)-α-[1-9*H*-flouren-9-yl)]methoylcarbonylamino]-2,4-dimethoxyphenoxyacetic acid (Fmoc-Rink-amide linker), 1-(4,4′-dimethyl-2,6-dioxocyclohexylidene)-3-ethyl (Dde), tetrakis (triphenylphosphine) palladium (0) (Pd(PPh_3_)_4_) and 2,3-diaminoproprionic acid (Dap).

### Peptide synthesis

All linear peptides were prepared by Fmoc-SPPS using a manually operated flow-chemistry apparatus, employing TentaGel^®^-S-NH_2_ resin at *ca.* 0.15 mmol scale. Disulfide cyclisation was performed post resin cleavage and global deprotection with *N*-chlorosuccinimide (1.2 equiv.) in a solution of MeCN/H_2_O (3:7, *v/v*) solution. Vinyl sulfide cyclisation was performed at physiological pH (7.4) in MeCN:MQ H_2_O (3:7, *v/v*) at 4 °C. See SI for further details.

### MIC (minimum inhibitory concentration) assay–bacteria

*Staphylococcus aureus* ATCC 29213, *Pseudomonas aeruginosa* (SVB-B9) (type strain) and *Escherichia coli* ATCC 25922 were grown in cation adjusted Mueller Hinton (MH) broth at 37°C with shaking (200 rpm). MIC assays were performed in accordance with the CLSI recommended protocols (Clinical and Laboratory Standards Institute, 2006, Performance standards for antimicrobial susceptibility testing; sixteenth informational supplement. CLSI document M100-S16, Wayne, PA., 2006). See SI for further details.

### MIC (minimum inhibitory concentration) assay–fungus

*Candida albicans* SC5314 (type strain) was grown using RPMI 1640 media (with glutamine and phenol red, without bicarbonate). MIC assays were performed in accordance with the CLSI recommended protocol (Clinical and Laboratory Standards Institute, 2006, Performance standards for antimicrobial susceptibility testing; sixteenth informational supplement. CLSI document M100-S16, Wayne, PA., 2006). See SI for further details.

### Circular dichroism (CD)

All CD spectra were recorded in either phosphate buffer (pH 7.4, 200 µM) or TFE:phosphate buffer (pH 7.4, 200 μM, 1:1, *v/v*) at a peptide concentration of 50 µM. All spectra were recorded at 20°C with a cuvette of 1 mm path length in a range from 180 nm to 260 nm at 0.5 nm intervals with a time-to-point of 0.5 s. Each spectrum was prepared from an average of five scans obtained with a 1 nm optical bandwidth. The baseline scans were collected with solvent alone, averaged, and then subtracted from sample scans.

## Results and discussion

### Initial peptide design

**FIGURE 3:**
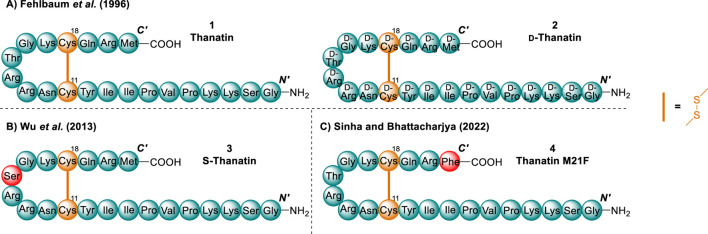
Summary of three of the key analogues developed over the years for thanatin (1). Key residue substitutions (red) and disulfides (orange) (Fehlbaum et al., 1996; Wu et al., 2008; Sinha and Bhattacharjya, 2022).

The chemical space surrounding thanatin has been explored extensively. Multiple series of analogues have been developed through truncations and substitutions to gain insight into its mechanism of action (MoA). Of the variety of analogues previously synthesised, the most poignant for the design of novel compounds active towards Gram-negative bacteria include: d-thanatin (**2**) by Fehlbaum et al. (1996), Ser substituted S-thanatin (**3**) by Wu et al*.* (2013) and thanatin M21F (**4**) by Sinha and Bhattacharjya (2022) (Figure 3). S-thanatin (**3**) and thanatin M21F (**4**) both presented examples of single amino acids substitutions whereby activity was improved or unaffected. Additionally, these substitutions replaced amino acids of increased synthetic difficulty, namely the *C-*terminal Met and Thr in position 15. While many more analogues of thanatin have been created, the analogues discussed are the most crucial with respect to our research (Lee et al., 2002; Hou et al., 2013; Zhou et al., 2018; Ma et al., 2019).

**FIGURE 4:**
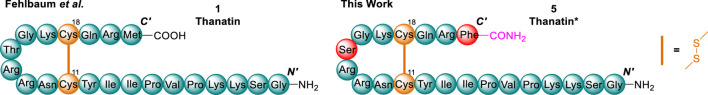
Chemical structures of native thanatin (1) and thanatin* (5). Amino acids substitutions (red), disulfide residues (orange), and *C-*terminal amidation (purple) highlighted.

We opted to implement prior knowledge from thanatin analogues to utilise an already optimised lead analogue in our studies, denoted herein as thanatin* (**5**). Thanatin* (**5**) synthesised and used as both a lead and reference compound within this work comprises two amino acid substitutions, adopted from analogues reported by Wu et al. (2013) and Sinha and Bhattacharjya (2022), and *C*-terminal amidation (Figure 4), namely the *C*-terminal Met and Thr in position 15 were substituted for Phe and Ser, respectively. These modifications were shown to improve or have little effect on activity, however, can significantly improve the ease of synthesis. Met is known to commonly form unwanted sulfoxide side products (Reusche and Thomas, 2021), and Thr often decreases amino acid coupling efficiency due to its more hindered β-branched structure (Humphrey et al., 1996). Furthermore the M21F substitution is known to enhance LptA binding affinity (Sinha et al., 2022), which we believe is a crucial interaction to mediate synergy with polymyxin B.

### Synthesis of thanatin analogue 5

#### Fast flow Fmoc-SPPS

**SCHEME 1:**
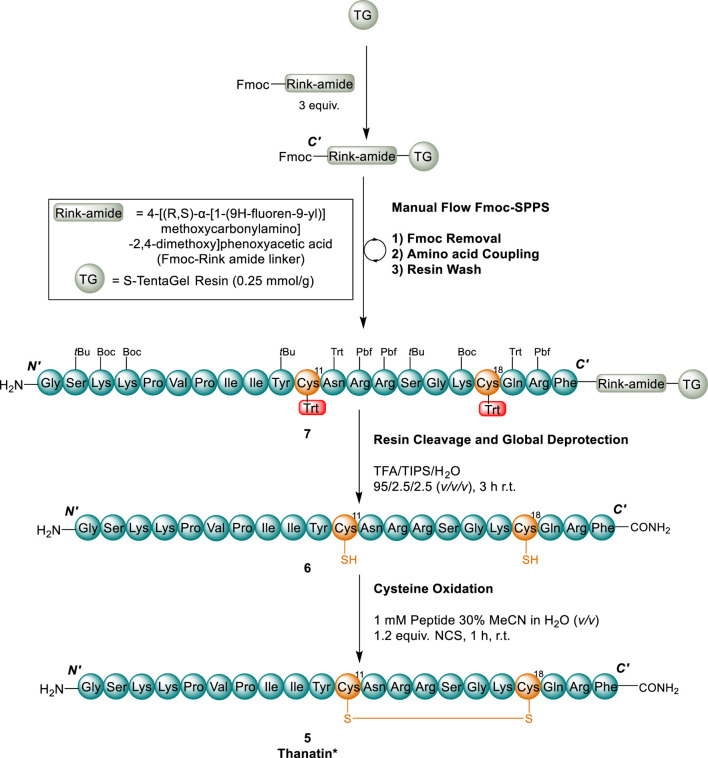
Synthesis of thanatin* (5). Cys residues and disulfides (orange), Cys (Trt) protecting groups (red). (1) 40 s flow (15 mL/min) 30% (*v/v*) piperidine in DMF; (2) AA coupling following protocol 2A for all non Cys AAs and protocol 2B for Cys residues (see SI); 2A) 30 s flow of AA coupling solution (0.3 M AA, 0.28 M HATU, 20 equiv. DIPEA) at 15 mL/min; 2B) 40 s flow of Cys (Trt) (0.3 M AA 0.28 M PyAOP, 20 equiv. *sym-*collidine) at 15 mL/min (3) 40 s flow of DMF at 15 mL/min. All flow reactions were performed at 65 °C.

For the synthesis of thanatin* (**5**), an early version of Pentelute and co-workers (2014) fast flow Fmoc-SPPS system was employed (Simon et al., 2014). The linear peptide was elongated on large particle size and high swelling TentaGel^®^-S-NH_2_ resin (600 mg, 0.15 mmol, 0.25 mmol/g), attached via a 4-[(R,S)-α-[1-9*H*-flouren-9-yl)]methoylcarbonylamino]-2,4-dimethoxyphenoxyacetic acid (Fmoc-Rink amide linker) to yield a *C*-terminal carboxamide functionality (Rink, 1987; Pires et al., 2014) in accordance with our previously optimised conditions ((Shepperson et al., 2021; Shepperson et al., 2024)) for the synthesis of Cys containing β-hairpin peptides (Scheme 1). Following complete elongation by flow chemistry to afford peptidyl resin **7**, the resin bound linear peptide was cleaved from the resin (Scheme 1) to yield linear peptide **6** in high purity (*ca.* 85%) (Supplementary Figure S15).

### Solution phase disulfide bond formation

With the linear peptide **6** in-hand, we envisaged an *N-*chlorosuccinimide (NCS) mediated solution-phase disulfide cyclisation as this approach is both mild and efficient, exhibiting quantitative conversion (Postma and Albericio, 2013). Accordingly, the linear peptide **5** was solubilised in a solution of 30% (*v/v*) acetonitrile in water at a concentration of 1 mM, to avoid oligomerization, and cyclisation initiated by the addition of a slight excess of *N*-chlorosuccinimide (1.2 equiv.) (Scheme 1).

Although, Postma and Albericio (2013) standardised this oxidation strategy and found the reaction to be quantitative and complete within 15 min, an extended period (1 h) was required to provide the best conversion (Supplementary Figure S16) to thanatin* (**5**). The reaction could be monitored by a noticeable 0.6 min shift in retention time (t_R_) on RP-HPLC and the product identity confirmed by an ESI-MS shift of 2 Daltons (Supplementary Figure S16). The need for an extended reaction may be due to the choice of aqueous solvent mixture (MeCN/H_2_O) in our work compared to dimethylformamide (DMF) used previously (Postma and Albericio, 2013). However, the choice of MeCN/H_2_O solvent system in this work enabled simple removal of the solvents by lyophilisation upon reaction completion, avoiding more complex work-up protocols for the removal of DMF. Thanatin* (**5**) was purified by semi-preparative RP-HPLC to yield a final isolated product in high purity (>95%, Supplementary Figures S1, S6, S11, S12) and moderate overall yield (6.9%, based on initial resin loading).

### Biological evaluation of analogue 5

Prior to preparing analogues in which the disulfide bond was modified with vinyl sulfide bridges, we sought to confirm the antimicrobial activity of the modified thanatin analogue **5** by minimum inhibitory concentration (MIC) assay (Table 1).

**TABLE 1:** Minimum Inhibitory Concentrations (MICs) of thanatin* (5) and similar analogues against a variety of pathogens.

Bacteria	Minimum inhibitory concentration (µM)					
	Thanatin^a^ ^,^ ^b^ (1)	S-Thanatin^a^ (3)	Thanatin M21F^b^ (4)	Thanatin* (5)	PMB	AMX
Gram-positive						
*S. aureus* ATCC 29213	-	-	-	>64	-	2
*S. aureus* ATCC 25923	8^b^	-	0.5	-	-	-
Gram-negative						
*E. coli* ATCC 25922	3.2^a^	1.6	-	1	0.125	-
*E. coli* BL21	0.5^b^	-	0.5	-	-	-
*P. aeruginosa* (SVB-B9)^c^	-	-	-	>64	0.125	-
*P. aeruginosa* ATCC 27853	1^b^	-	0.5	-	-	-

^a^ Values cited from Wu et al. (2013).

^b^ Values cited from Sinha et al. (2022).

^c^ Type strain.

To our surprise, thanatin* (**5**) exhibited no activity against Gram-positive bacteria (*S. aureus*) and varied activity against Gram-negative bacteria (*P. aeruginosa* and *E. coli*). Towards *P. aeruginosa,* thanatin* (**5**) was inactive within the range tested (MIC >64 µM). However, against *E. coli*, thanatin* (**5**) was highly active with a MIC of 1 µM. On comparison of these results with those presented in literature for similar analogues, our analogue, thanatin* (**5**), was at least equipotent towards *E. coli*. Of note, the analogues S-thanatin (**3**) and thanatin M21F (**4**) (Figure 3) were both reported to be highly active towards *S. aureus* and *P. aeruginosa*, for which our analogue thanatin* (**5**) exhibited no activity (Table 1). Our testing employed cation adjusted media to better mimic physiological conditions, while the previously reported analogues were tested in non-cation adjusted media (Wu et al., 2008; Sinha and Bhattacharjya, 2022); the literature results are included for comparison (Table 1). It is well-documented that divalent cations, present under physiological conditions, can antagonise the interactions of cationic AMP’s with bacterial membranes resulting in decreased potency (Goldman et al., 1997; Wiegand et al., 2008; Smart et al., 2017; Ma et al., 2019). Specifically, previous research has observed similarly decreased activity for thanatin towards *P. aeruginosa* in cation adjusted media (Fehlbaum et al., 1996; Cirioni et al., 2011), This finding is of key importance if these were to be developed as drugs. Pleasingly, our analogues appear to have an even greater degree of selectivity towards *E. coli* over *P. aeruginosa* (>64-fold) than that of those previously reported.

Thanatin and analogues are known to interact with LPS, forming a unique amphipathic dimer that is thought to offer selectivity towards the Gram-negative outer membrane (Sinha et al., 2020; Sinha and Bhattacharjya, 2022). These interactions lead to the displacement of divalent cations from the outer membrane, resulting in membrane disruption, which ultimately provides thanatin with access to its additional protein target (LptA) in the periplasmic space (Ma et al., 2019; Sinha et al., 2022). Given the important role of divalent cation displacement in the mechanism of action of thanatin, and their role in Gram-negative outer membrane structures (Shephard et al., 2008), it is not surprising that cation concentrations can alter thanatin derivative’s activity profiles. More surprising is the apparent deviation of this effect between Gram-negative species. Although *P. aeruginosa* is well documented to possess a an exceptionally impermeable outer membrane, believed to offer less than 10% the permeability of *E. coli* (Hancock, 1998), the exact molecular differences in the membrane structures responsible for the differing activity of our thanatin analogues between these two species of Gram-negative pathogens is unclear. The diminished sensitivity of *P. aeruginosa* in the presence of divalent cations suggests thanatin may interact with the LPS of the *P. aeruginosa* membrane less effectively than for *E. coli*, resulting in a reduced capacity to compete for cation binding sites. Despite the inactivity of thanatin analogue **5** towards *P. aeruginosa*, the >64 fold selectivity towards *E. coli* is ideal in our search for new and effective strategies to treat Gram-negative pathogens, as partial or reduced activity (as observed for other thanatin derivatives) towards certain species may pose the greatest risk for resistance to develop. Narrow spectrum antibiotics with high and directed potency toward specific bacteria have been shown to develop resistance at a reduced rate than those exhibiting broad spectrums of activity with their potency varying towards different pathogens (Melander et al., 2018; Alm and Lahiri, 2020; Ueda et al., 2023). Furthermore, the potent activity of thanatin* (**5**) towards *E. coli* is crucial. *E. coli* is an ever-increasing global health risk, with drug-resistant *E. coli* declared as critical priority (Tacconelli et al., 2018; Mughini-Gras et al., 2019). It has been identified as one of the largest contributors to diarrhoeal death, and with the largest effected communities often unable avoid infection, effective treatments are urgently required (Erb et al., 2007; Ramatla et al., 2023).

### Synergy with polymyxin B

Having confirmed the selective and potent activity of thanatin* (**5**) towards *E. coli*, this AMP was assayed for synergy with polymyxin B towards *E coli* by use of a checkerboard assay. Synergy assays were performed with three repeats to determine the average fractional inhibitory concentration indices (FICI) (Odds, 2003). FICs were calculated from Equation 1 and defined as follows; synergy occurred at an FIC ≤0.5, antagonism at FIC >4.0 and an additive effect in the range 0.5–4.0. Thanatin* (**5**) exhibited synergy with polymyxin B, demonstrating an average FICI of 0.347 with a standard deviation of 0.03 (Supplementary Figure S13). Given thanatin (1) is known to inhibit LPS transport by binding LptA, we postulate this synergy may result from of an inhibitory interaction on the Lpt system acting cooperatively with polymyxin B, similar to the synergy recently documented for LptD binder murepavidin (Dale et al., 2018). In fact, a thanatin analogue, S-thanatin, was reported in 2011 (Cirioni et al., 2011) to have a positive interaction with the similar lipopeptide antibiotic colistin (polymyxin E) (Kwa et al., 2007), with an FICI of 0.46 for *P. aeruginosa*, despite its poor potency towards this pathogen (MIC = 16 μg/mL). The synergy with polymyxin B that we report towards *E. coli* is particularly valuable due to the high potency of thanatin analogue **5** (1 µM MIC, *ca.* 2.5 μg/mL). The previous work has received surprisingly little attention, despite also reporting *in vivo* efficacy of the combination and a reduction in the emergence of polymyxin resistance, possibly due to the lack of evidence or speculation as to the underlying mechanism. However, with the rising global threat of AMR and increasing necessity of polymyxin antibiotics as a last-line of defence, these finding are increasingly valuable. Importantly, we highlight that the molecular mechanism for this synergy is likely to be the result of thanatin analogues’ ability to disrupt LPS transport, which is a mechanism only more recently elucidated for thanatin. Furthermore, the potential for LPS disruption to synergise with polymyxins is also only more recently established from findings with the LptD inhibitor, murepavidin (Sabnis et al., 2021). With the likely mechanism of synergy now understood, these findings pave the way for further elucidation of molecular mechanisms and to progress thanatin analogues or LptA binders as synergistic agents to compliment polymyxin antibiotics in the treatment of drug-resistant *E. coli*, noting that murepavidin is active only towards *P. aeruginosa* (Dale et al., 2018).

$$FIC\;Index=FIC\;A+FIC\;B=\frac{{MIC}_{A}^{Combination}}{{MIC}_{A}^{Alone}}+\frac{{MIC}_{B}^{Combination}}{{MIC}_{B}^{Alone}}$$ (1)

Equation 1 to determine FICs for two antibacterial agents

### Vinyl sulfide library design

Encouraged by potent and selective antimicrobial activity of thanatin* (**5**) towards *E. coli*, and its synergistic activity with polymyxin B, we were curious if these properties would be retained upon disulfide bond replacement. We envisioned implementing thia-Michael addition between the sidechain thiol of a Cys residue and an allenamide to mediate a facile cyclisation yielding a vinyl sulfide that is not prone to reductive cleavage like the native disulfide (Abbas et al., 2014). This approach was recently implemented on oxytocin by Cameron et al. (2020) as a model compound and most recently to prepare analogues of the antimicrobial β-hairpin, capitellacin, in a similar manner to that which we envisaged for thanatin* (**5**). In addition to a facile cyclisation, this strategy offers versatility with respect to overall bridge length and provides greater flexibility to the design of an analogue library.

**FIGURE 5:**
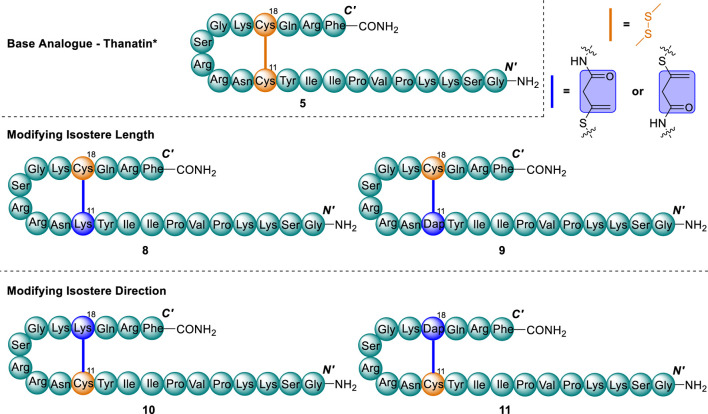
Analogue library of thanatin* (5) and vinyl sulfide analogues (8–11). Cys residues and disulfide (orange) and substituted Cys residue and vinyl sulfide bridge (blue).

Taking the thanatin*(**5**) scaffold, a series of four vinyl sulfide analogues were designed by varying both the length and direction of the newly introduced asymmetrical bridge. (Figure 5). The overall bridge length and direction of the vinyl sulfide was controlled via the replacement of a single Cys residue of the native disulfide with either 2,3-diaminoproprionic acid (Dap) or Lys residue, upon which an allenamide handle could be introduced. Upon thia-Michael mediated cyclisation, these replacements yield a bridge of seven or ten atoms total, compared with four for the native disulfide. Introducing bridges of increased length has previously been demonstrated as an effective strategy to modify disulfides in β-hairpin AMPs, and in some cases has improved the therapeutic index (Fázio et al., 2006; Holland-Nell and Meldal, 2011; Cui et al., 2013).

### Synthesis of vinyl sulfide analogues, 8–11

**SCHEME 2:**
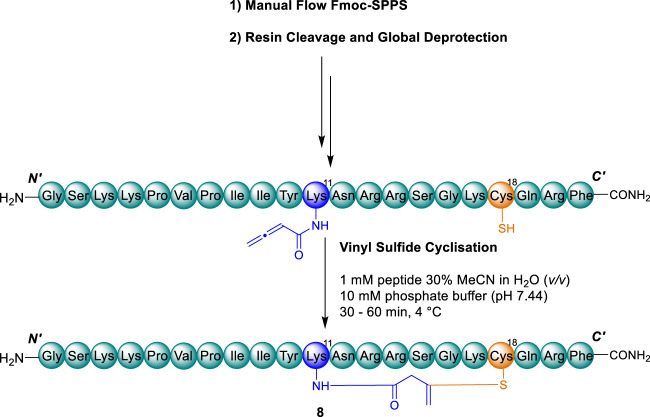
Representative synthetic scheme for cyclisation of vinyl sulfide analogues 8–11. Cys residues and disulfide (orange) and substituted Cys residue and vinyl sulfide bridge (blue).

The linear precursors of vinyl sulfide analogues 8–11 were prepared by fast flow SPPS analogously to thanatin* (**5**), incorporating the required Cys residue substitutions. In order to enable site selective introduction of the allenamidyl handle by the coupling of 3-butynoic acid (3-BA), orthogonal sidechain protection was introduced to the corresponding Lys or Dap residue (Supplementary Scheme S1, S2). We have previously reported 1-(4,4′-dimethyl-2,6-dioxocyclohexylidene)-3-ethyl (Dde) protection to be unsuitable for Dap residues during flow chemistry under high temperature. This protection strategy led to undesired migration, however, this could be avoided by use of the *N-*allyloxycarbonyl (*N* ^*β*^ *-*Alloc) group. Accordingly, Alloc protection was implemented for Dap residues, and Dde protection implemented for Lys residues, where these respective amine side chain amino acids were substituted in place of Cys (Shepperson et al., 2024). Following complete assembly of the linear peptides on-resin, Fmoc-protection of the terminal residue was exchanged for Boc where Dde protection was present and the orthogonal protection removed from the amino-sidechains of Dap/Lys under standard conditions. Dde was removed in-flow by treatment with hydrazine hydrate (Shepperson et al., 2024) and Alloc removed by batch-wise treatment with (Shepperson et al., 2024). To the respective liberated free amino groups of Dap/Lys, 3-BA was coupled to introduce the allenamidyl handle in accordance with our previously optimised protocol (Cameron et al., 2020; Shepperson et al., 2024). The resultant peptides were liberated from the resin to afford the desired linear precursors of peptides 8–11 for cyclisation (Scheme 2).

Implementing the conditions we previously developed (Cameron et al., 2020) the linear precursors of the desired peptides **8**–**11** were cyclised by thia-Michael addition to afford the desired vinyl sulfide bridge analogues, simply by addition of phosphate buffer (10 mM, pH ∼7.4) at 4 °C (Scheme 2). Reaction progress was monitored by RP-HPLC with the addition of 5,5-dithio-bis-(2-nitrobenzoic acid) (DTNB) employed for reaction quenching (Supplementary Scheme S3) (Cameron et al., 2020; Shepperson et al., 2024). Pleasingly, the reactions progressed with high conversion to the desired cyclised peptides **8–11** (80%–90%, as indicated by RP-HPLC and ESI-MS) within 30–60 min. Upon addition of DTNB to the reaction solution, undesired side products bearing a free thiol were shifted to later retention times, allowing for straightforward purification by semi-preparative RP-HPLC (Supplementary Figures S2–S5, S7–S10). The free thiol test employing DTNB was validated with the linear precursor of peptide **9** (Supplementary Figure S17) which also exhibited a significant retention shift upon mixed-disulfide adduct formation (Supplementary Scheme S3).

Interestingly, vinyl sulfide analogues (**8–11**) of thanatin (**5**) eluted earlier than our disulfide analogue of thanatin (**5**) on RP-HPLC (Supplementary Table S1). Additionally, Lys bridged analogues were found to elute earlier than Dap bridged analogues (Supplementary Table S1), suggesting they present a less hydrophobic structure.

### Antimicrobial activity of analogues 8–11

Thanatin vinyl sulfide analogues **8–11** were tested for antimicrobial activity against a small series of bacterial and fungal pathogens, including *P. aeruginosa, E. coli*, *S. aureus* and *C. albicans* (Table 2). For antibacterial testing polymyxin B (PMB) or amoxicillin (AMX) were used as controls and for fungal testing amphotericin B (AMB) was employed.

**TABLE 2:** Minimum Inhibitory Concentrations (MICs) of thanatin* (5) and analogues (8–11) towards a variety of pathogens.

Bacteria	Minimum inhibitory concentration (µM)								
	1 (Thanatin)^a^	5 (Thanatin*)	8	9	10	11	PMB	AMX	AMB
Gram-positive									
*S. aureus* ATCC 29213	-	>64	>64	>64	>64	>64	-	2	-
*S. aureus* ATCC 25923	8	-	-	-	-	-	-	-	-
Gram-negative									
*E. coli* ATCC 25922	0.5	1	4	16	4	8	0.125	-	-
*P. aeruginosa* (SVB-B9)^b^	-	>64	>64	>64	>64	>64	0.125	-	-
*P. aeruginosa* PAO1	2	-	-	-	-	-	-	-	-
Fungi									
*C. albicans*	-	>64	>64	>64	>64	>64	-	-	1

^a^ Values cited from Sinha et al. (2022).

^b^ Type strain.

Pleasingly, vinyl sulfide analogues **8–11** retained antimicrobial selectivity towards *E. coli*. However, the potency of the analogues was reduced to varying extents. Lys substituted analogues (**8** and **10**) retained the greatest potency, each with an MIC of 4 µM towards *E. coli*, while Dap analogue **9** (MIC 16 µM) demonstrated a 16-fold reduction in potency compared to the thanatin* (**5**). Neither thanatin* (**5**) nor the vinyl sulfide analogues (**8–11**) exhibited antifungal activity towards *C. albicans*, despite the documented antifungal activity of thanatin (**1**). The selective antimicrobial potency retained by analogues **8** and **10** was promising as it suggested the viability of vinyl sulfides for the substitution of disulfides in AMPs and perhaps further bioactive peptides. These results similarly coincide with previous work performed when replacing the disulfide bridges of similar β-hairpin peptides, whereby increased bridge flexibility inevitable leads to slight reduction in activity (Fázio et al., 2006; Holland-Nell and Meldal, 2011; Chen et al., 2020; Shepperson et al., 2024). This result suggests further exploration of different bridge surrogates could be a useful strategy in developing therapeutic AMPs.

### Synergism with polymyxin B

Curious to investigate whether synergy with polymyxin B would be retained following replacement of the disulfide, vinyl sulfide **8** was subjected to a checkerboard assay towards *E. coli* with polymyxin B (Odds, 2003). Delightfully, analogue **8** retained a synergistic effect with polymyxin B, demonstrating an average FICI of 0.429 (SD 0.07) (Supplementary Figure S14). The observed synergy suggests that inhibition of the Lpt system in Gram-negative bacteria is retained as an MoA upon disulfide substitution in analogue **8**, despite the change RP-HPLC retention time suggesting a structural change may have occurred. These results further validate the vinyl sulfide as a simple synthetic approach to a non-reducible bridge alternative.

### Circular dichroism investigation of thanatin* (5) and analogues 8–11

**FIGURE 6:**
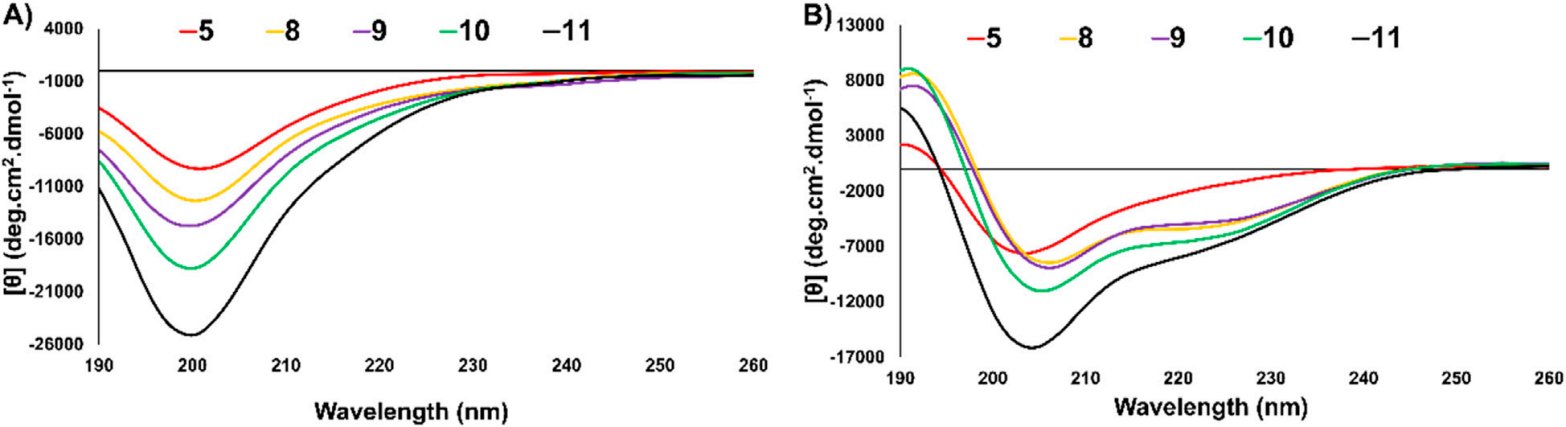
**(A)** CD spectra overlay of thanatin* (5) and analogues (8–11) at 50 µM in 200 µM sodium phosphate buffer (pH ∼7.4, 20°C). **(B)** CD spectra overlay of thanatin* (5) and analogues (8–11) at 50 µM in TFE:200 µM sodium phosphate buffer 1:1 (pH ∼7.4, 20°C).

To examine the structure of thanatin* (**5**) and the vinyl sulfide analogues (**8–11**), CD spectra were recorded in both phosphate buffer and a TFE:phosphate buffer (1:1, *v/v*) mixture (Figure 6). To investigate the structure of the peptide at biological pH (200 µM buffer) and an environment that stabilises the secondary structure of peptides (TFE: buffer; 1:1, *v/v*), representative of interaction with biological membranes (Nelson and Kallenbach, 1986).

As thanatin (**1**) has been previously studied by NMR, its secondary structure has been defined as a β-hairpin. It has large β-sheet regions (residues 8–21) and an *N-*terminal flexible portion (residues 1–7) exhibiting random coil elements. Additionally, the β-sheet region of the peptide is relatively small, contributing less to the overall ellipticity of the entire peptide. Due to these conflicting elements, the CD spectra of thanatin (**1**) is not representative of a particular secondary structure. Instead, the CD spectra can be used as a fingerprint of the molecules secondary structure, which has been previously defined by NMR (Lee et al., 2002; Ma et al., 2016), enabling comparison of analogue **5** and **8**–**11** to indentify changes in secondary structure.

When thanatin* (**5**) and vinyl sulfide analogues (**8–11**) were examined in pH 7.4 buffer (Figure 6A) all peptides exhibited spectra representative of random coil, all of which were highly similar to one another and the literature data for native thanatin (**1**). Each analogue exhibited negative maxima at ∼200 nm (Lee et al., 2002). In the secondary structure favouring environment (TFE:phosphate buffer; 1:1, *v/v*, Figure 6B) thanatin* (**5**) and analogues (**8–11**) exhibited slightly more defined secondary structures, in agreement with the literature (Venyaminov and Yang, 1996; Lee et al., 2002; Ma et al., 2016). Positive maxima were observed between 190 nm–195 nm for all peptides. Additionally, vinyl sulfide analogues **8–11** exhibited a negative maxima between 200 nm–205 nm with **8–10** exhibiting a second smaller negative maxima at ∼220 nm. Collectively, CD experiments evidenced minimal change in secondary structure between the disulfide bridged thanatin* (**5**) and vinyl sulfide analogues **8**–**11**, despite the changes in RP-HPLC retention time noted earlier. These results suggested that a replacement of disulfide by vinyl sulfide does not drastically alter the secondary structure of thanatin* (**5**). Furthermore, the length and direction of the vinyl sulfide bridge appears to have little effect on the secondary structure despite its more substantial impact on antimicrobial activity. Interestingly, in our previous work (Shepperson et al., 2024), changing length and direction of vinyl sulfide bridges had a more drastic impact on the secondary structure of capitellacin analogues, suggesting the effect of disulfide replacement with vinyl sulfides is scaffold dependent.

## Conclusion

This study presents the first investigation into the potential of thanatin analogues as synergistic agents in combination with polymyxin B, and the replacement of the disulfide bridge in thanatin. Several analogues of the antimicrobial β-hairpin peptide thanatin (**1**) were prepared by chemical synthesis using flow chemistry. A simple analogue of thanatin (**1**) carrying C-terminal amidation and two residue substitutions (thanatin* **5**), demonstrated potent and selective inhibitory activity towards *E. coli*. This peptide demonstrated a synergistic effect with polymyxin B, a last line of defence antibiotic for Gram-negative infections. Given thanatin (**1**), is known to bind to LptA and inhibit LPS transport in Gram-negative bacteria, we postulate this mechanism may be responsible for the observed synergy.

Replacement of the native disulfide bridge in this analogue with vinyl sulfide bridge mimetics, prepared through thia-Michael addition of a Cys thiol to an allenamide handle, afforded four analogues (**8–11**). These analogues retained similar hairpin conformations as evidenced by CD spectroscopy and two compounds retained substantial selective antimicrobial activity towards *E. coli,* highlighting suitability of the vinyl sulfide as a versatile and facile strategy to replace reduction prone disulfide bridges. Furthermore, analogue **8** was shown to retain synergistic activity with polymyxin B.

The narrow spectrum antimicrobial activity demonstrated by these peptides is promising for the development of new antibiotics for the treatment of *E. coli* with reduced impact on the host microbiome and more limited development of resistance. These findings not only highlight the potential of thanatin* (**5**) and our vinyl sulfide analogues (**8–11**) as standalone antimicrobial agents, but also as promising candidates for combination therapies. Employing combinatorial treatments could enhance the potential for polymyxin B use by reducing the need for nephrotoxic doses and pave the way for innovative approaches to tackling antibiotic resistance. In particular, the ability of highly potent thanatin analogues to synergise with polymyxin antibiotics towards *E. coli* at low concentrations is promising for the development of strategies to combat AMR, particularly in light of the highly anticipated new generation of polymyxins in the development pipeline.

## Data Availability

The original contributions presented in the study are included in the article/Supplementary Material, further inquiries can be directed to the corresponding authors.
