# Supplementary material for: Thanatin and vinyl sulfide analogues as narrow spectrum antimicrobial peptides that synergise with polymyxin B
Source: Front Pharmacol. 2024 Nov 5;15:1487338. doi: 10.3389/fphar.2024.1487338 (PMC11573584; doi:10.3389/fphar.2024.1487338)
Supplement: Supplementary file 1 [file DataSheet1.pdf]

Thanatin and Vinyl Sulfide Analogues as Narrow Spectrum Antimicrobial Peptides that Synergise with Polymyxin B

Oscar A. Shepperson[1,2,3], Paul W. R. Harris[1,2,3*], Margaret A. Brimble[1,2,3*], and Alan J. Cameron[1,2,3,*]

[1]The University of Auckland, School of Chemical Sciences, 23 Symonds St., Auckland 1010, New Zealand.

[2]The University of Auckland, School of Biological Sciences, 3A Symonds St., Auckland 1010, New Zealand.

[3]Maurice Wilkins Centre for Molecular Bio-Discovery. The University of Auckland, 3A Symonds St. Auckland 1010, New Zealand.

***Correspondence:**
Alan Cameron
Alan.cameron@auckland.ac.nz

Margaret Brimble

M.brimble@auckland.ac.nz

Paul Harris

Paul.harris@auckland.ac.nz

Supplementary Material

1 General Information

All the reagents purchased from commercial sources were reagent grade and were used without further purification. Solvents for peptide synthesis and RP-HPLC were purchased as synthesis grade and HPLC grade, respectively.

4-[(*R*,*S*)-α-[1-(9*H*-Fluoren-9-yl)]methoxycarbonylamino]-2,4-dimethoxy]phenoxyacetic acid (Fmoc-Rink amide linker), polymyxin B sulphate (PMB), tetrakis(triphenylphosphine)palladium(0) ($Pd(PPh_3)_4$) and *N*-ethoxycarbonyl-2-ethoxy-1,2-dihydroquinoline (EEDQ) were purchased from AK Scientific (Union City, CA, USA). (7-Azabenzotriazol-1-yloxy)trispyrrolidinophosphonium hexafluorophosphate (PyAOP) was purchased from Aapptec (Louisville, KY, USA). *N,N*-diisopropylethylamine (DIPEA), 2,4,6-trimethylpyridine (*sym*-collidine), triisopropylsilane (TIPS), piperidine, 3-butynoic acid (3-BA), hydrazine, 5,5-dithio-bis-(2-nitrobenzoic acid) (DTNB, Ellman's Reagent), amphotericin B (AMB), phosphate buffer saline (PBS), phenylsilane ($PhSiH_3$), sodium diethyldithiocarbamate, trifluoroethanol (TFE) and amoxicillin (AMX) were purchased from Sigma–Aldrich (St Louis, MO, USA). Diethyl ether (Et_2O) was purchased from Avantor Performance Materials (Center Valley, USA). Trifluoroacetic acid (TFA) was purchased from Oakwood Chemicals (Estill, SC, USA). Oxoid Mueller Hinton Broth (MHB), *N,N*-dimethylformamide (DMF; AR grade) and acetonitrile (MeCN, HPLC grade) were purchased from Thermo Scientific (Hampshire, NH, USA). Dichloromethane (CH_2Cl_2) was purchased from ECP Limited (Auckland, New Zealand). TentaGel®-S-NH_2 resin was purchased from RAPP Polymere (Tubingen, Germany). Polypropylene 96-well flat bottom plates were purchased from Greiner Bio-One (Kremsmünster, Austria). Milli-Q high purity deionised water (MQ H_2O) was available from a Sartorius Arium® Pro Ultrapure Water System from Sartorius Stedim Biotech (Gottingen, Germany). 1-[Bis(dimethylamino)methylene]-1*H*-1,2,3-triazolo[4,5-*b*]pyridinium 3-oxide hexafluorophosphate (HATU), Boc-Gly-OH (Boc = *tert*-butyloxycabonyl), and all Fmoc-amino acids were purchased from CS Bio Ltd (Shanghai, China). The supplied amino-acids were side chain protected as follows: Fmoc-Arg(Pdf)-OH (Pbf = 2,2,4,6,7-pentamethyldihydrobenzofuran-5-sulfonyl), Fmoc-Asn(Trt)-OH, (Trt = triphenylmethyl), Fmoc-Cys(Trt)-OH, Fmoc-Ser(*t*Bu)-OH (*t*Bu = *tert*-butyl), Fmoc-Trp(Boc)-OH, Fmoc-Lys(Boc)-OH, Fmoc-Lys(Dde)-OH (Dde = 1-(4,4'-dimethyl-2,6-dioxocyclohexylidene)-3-ethyl), Fmoc-Gln(Trt)-OH, Fmoc-Dap(N^{β}-Alloc)-OH (N^{β}-Alloc = *N*-Allyloxycarbonyl) and Fmoc-Tyr(*t*Bu)-OH).

Analytical reverse phase high-performance liquid chromatography (RP-HPLC) was performed on a Waters (Waltham, MA, USA) Alliance analytical HPLC equipped with a Phenomenex (Torrance, CA, USA) Luna C18 column (100 A, 5 μm, 4.6 mm x 250 mm) operated at room temperature, with chromatograms recorded at 214 nm and 254 nm. Semi-preparative RP-HPLC was performed on a Waters 1525 Binary HPLC pump equipped with a Waters 2489 UV/visible detector (214 nm) using a Phenomenex Luna C18 semi-prep column (100 Å, 5 μm, 250 mm x 10 mm). For both analytical and semi-preparative RP-HPLC, solvents used were as follows: solvent A = 0.1% TFA in water (MQ H_2O) and solvent B = 0.1% TFA in MeCN. For analytical RP-HPLC the gradient employed was 5-45% of solvent B over 40 minutes at flow rate of 1 mL/min, unless specified otherwise. For semi-preparative RP-HPLC gradients were adjusted as indicated in the experimental procedures, according to elution times and peak profiles obtained during analytical analysis. Flow setup specifications were as follow; VICI M series pump (Model M50, P/N CP3A-8182-F2), VICI 10 port switching valve (Model C25, P/N C25-6180EUHB), 1/8” OD tubing between switching valve and pump and 1/16” OD tubing for

all other setup areas. The reaction vessel was manufactured according to the specifications as outlined by Simon *et al.* (2014).

High-resolution mass spectrometry (HRMS) was performed with a Bruker (Billerica, MA, USA) micrOTOFQ mass spectrometer by using electrospray ionisation (ESI) in the positive mode at a nominal accelerating voltage of 70 eV. Low-resolution mass spectrometry was performed on a Waters Quattro micro–API Mass Spectrometer in ESI positive mode.

2 General Methods

2.1 Method 1: Resin Functionalisation for *C*-terminal carboxamide peptides

Manual flow synthesis was performed on a *ca.* 0.15 mmol scale. Loading of the Fmoc-Rink amide linker (324 mg, 0.60 mmol, 4 equiv.) to TentaGel®-S-NH_2 resin (600 mg, 0.15 mmol, 0.25 mmol/g) was undertaken prior to flow-SPPS using HATU (216 mg, 0.57 mmol, 3.8 equiv.) and DIPEA (261 µL, mmol, 10 equiv.) in DMF for 3 h at r.t. Following linker coupling the resin was washed with DMF (3 x 5 mL) and CH_2Cl_2 (3 x 5 mL). Peptide elongation was performed by manual flow synthesis (**Method 2**).

2.2 Method 2: Manual Flow Synthesis of Linear Peptides

During manual flow synthesis, the deprotection solution for removal of the N^{α}-Fmoc protecting group consisted of 30% piperidine (*v/v)* in DMF. Prior to beginning the synthesis, amino acid coupling solutions were freshly prepared in DMF at a concentration of 0.30 M of amino acid (1.5 mmol, 10 equiv.) and 0.28 M of coupling reagent (HATU, 1.43 mmol, 9.5 equiv.). Prior to each amino acid coupling, DIPEA (522 µL, 3.00 mmol, 20 equiv.) was added to pre-activate the coupling solution 10 seconds prior to delivery to the on-resin peptide bearing a free N^{α}-amino group.

Reagents were delivered via a 5 ml stainless steel heating loop submerged in a water bath (65 °C), to the stainless-steel reaction vessel, also submerged in a water bath (65 °C), containing the functionalised peptidyl resin. All reagents were delivered at a constant flow rate of 15 mL/min unless specified otherwise. N.B; *Sub-Method 3.1***.**

The manual flow synthesis began with pre-washing the resin with DMF (300 s, 75 mL) and performing the initial deprotection of the Fmoc protected amino acid or Rink amide linker, as appropriate (40 s, 10 mL). Following Fmoc-Rink amide deprotection, the resin was washed again with DMF (40 s, 10 mL).

Amino acids were coupled in order from the *C*- to *N*-terminus via four repeating steps of; (**1**) coupling, (**2a/b**) washing, (**3**) deprotection and (**4**) washing; outlined as follows:

(1) The activated amino acid solution is coupled to the free *N*-terminus of the previous residue/linker of the peptidyl resin (25-30 s, 6.6 mL).

(2)

a. The delivery line for the activated amino acid solution is washed by drawing DMF (15 s, 3.8 mL).

b. The peptidyl resin is further washed with DMF, removing any residual activated amino acid coupling solution (40 s, 10 mL).

(3) The N^{α}-Fmoc protecting group is removed from the newly coupled amino acid with the deprotection solution of 30% piperidine in DMF (*v/v*) (40 s, 10 mL).

(4) Peptidyl resin is washed, removing any residual deprotection solution (40 s, 10 mL).

Following the completion of peptide sequence the N^{α}-Fmoc protected resin bound peptide was washed with DMF (300 s, 75 mL, 65 °C), to remove any possible remaining residual reagents.

2.2.1 Sub-Method 2.1 – Cys coupling

The coupling of the Fmoc-Cys(Trt)-OH/Fmoc-Cys(Acm)-OH residues (0.30 M, 1.50 mmol, 10 equiv.) was performed with PyAOP (0.28 M, 1.43 mmol 9.5 equiv.) and *sym*-collidine (396.4 µL, 3.00 mmol, 20 equiv.) at a flow rate of 15 mL/min at 65 °C.

2.3 Method 3: In-flow Orthogonal Removal of Dde from Lys by Hydrazine

The resin was treated with a solution of 2% hydrazine (*v/v*) in DMF (3 x 20 s, 65 °C) at a flow rate of 15 mL/min. Following the final treatment, the resin bound peptide was washed with DMF (300 s, 75 mL, 65 °C) to remove any residual reagents.

2.4 Method 4: Orthogonal Removal of N^{β}-Alloc from Dap by Palladium (0)

The protected peptidyl resin bearing the N^{β}-Alloc protected Dap residue was swollen in dry CH_2Cl_2 (dried over molecular sieves). To the swollen resin was added a mixture of $Pd(PPh_3)_4$ (231.1 mg, 0.60 mmol, 4 equiv.) and $PhSiH_3$ (185 µL, 1.50 mmol, 10 equiv.) in dry CH_2Cl_2 and the reaction mixture agitated under an inert atmosphere, in the dark for 2 h at r.t. Following reaction completion, $Pd(PPh_3)_4$ was quenched with treatments of 0.5% sodium diethyldithiocarbamate (*w/v*) in DMF (3 x 3 min) washed with DMF (3 x 5 mL), CH_2Cl_2 (3 x 5 mL) and dried under vacuum (Abdel Monaim et al., 2017).

2.5 Method 5: 3-Butynoic Acid Coupling

The protected peptidyl resin containing a free side chain amino group was swollen in dry CH_2Cl_2 (dried over molecular sieves). To the swollen peptidyl resin was added a mixture of 3-butynoic acid (126 mg, 1.5 mmol, 10 equiv.), EEDQ (352 mg, 1.43 mmol, 9.5 equiv.) and *sym*-collidine (178 µL, 1.35 mmol, 9 equiv.) in dry CH_2Cl_2 and the reaction mixture agitated at r.t. for 2 h (Cameron et al., 2020). Following coupling, the peptidyl resin was thoroughly washed with CH_2Cl_2 (3 x 5 mL), DMF (3 x 5 mL) and again with CH_2Cl_2 (3 x 5 mL) and dried under vacuum.

2.6 Method 6: Resin Cleavage and Global Deprotection

The resin-bound peptide was treated with a cleavage cocktail of TFA/H_2O/TIPS (10 mL, 9.5/2.5/2.5, *v/v/v*) and agitated (3 h, r.t.). The cleavage solution was separated from the resin and its volume reduced under a flow of N_2. Cold Et_2O (45 mL) was used to precipitate the peptide. The pellet was isolated by centrifugation, washed once more with cold Et_2O (45 mL), dissolved in MeCN/MQ H_2O (3:7, *v/v*) and lyophilised.

2.7 Method 7: Solution Phase Disulfide Formation using *N*-chlorosuccinimide

The completely liberated peptide was solubilised in a solution of MeCN/MQ H_2O (3:7, *v/v*) at a concentration of 1 mM and treated with a slightly greater stoichiometric amount of NCS (1.2 equiv.) for 60 – 120 mins at r.t. (Postma and Albericio, 2013). Following reaction completion, as determined by RP-HPLC, the peptidyl solution was lyophilised.

2.8 Method 8: Solution Phase Vinyl Sulfide Cyclisation

The crude allenamide modified peptide was dissolved in a solution of MeCN/MQ H_2O (3:7, *v/v*) at 4 °C. To the solution was added 200 mM phosphate buffer (1/20 total vol., pH ~7.4, 4 °C) to maintain the final solution at a concentration of 1 mM peptide and 10 mM buffer. The solution was maintained at 4 °C for 3 − 60 min, reaction progression monitored by RP-HPLC, and quenched upon completion with DTNB according to **Method 9.**

2.9 Method 9: Quenching of Unreacted Thiols by DTNB

To the vinyl sulfide cyclisation solution was added a concentrated solution of DTNB (5 mg/mL, 4 °C, *ca.* 2 equiv.) in MQ H_2O. The solution was mixed for 30 – 60 mins at 4 °C, monitored by RP-HPLC, and upon observed reaction completion lyophilised.

3 Protocol For Synthesis of Peptides 5, 8 – 11

Thanatin* (**5**)

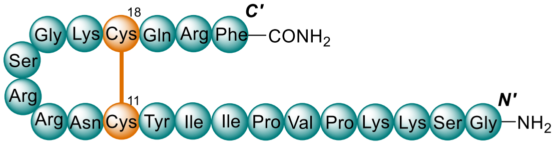

Following the functionalisation of the TentaGel®-S-NH_2 resin with Fmoc-Rink amide linker via **Method 1**, thanatin* (**5**) was synthesised by manual flow synthesis according to **Method 2** employing **Sub-Method 2.1** for the introduction of Cys(Trt) at positions 11 and 18. The final linear peptide was liberated from the resin and protecting groups simultaneously removed following **Method 6**, to afford the crude linear peptide (232 mg, 64% yield [based on initial resin loading]). The disulfide bond was formed in solution via **Method 7**, (1 mM peptide, 15.2 mg NCS, 0.114 mmol, 1.2 equiv.) to yield the final peptide, thanatin* (**5**), at approximately 85% purity.

The crude peptide (112 mg), thanatin* (**5**), was dissolved in a solution of MeCN/MQ (1:9 , *v/v*) H_2O at a concentration of ~12 mg/mL and purified (7 x ~2 mL injections) with a Phenomenex Luna C18 column (100 Å, 5 µm, 250 mm x 10 mm), employing a gradient with an isocratic phase of 5%B for 5 min, follow by a linear gradient to 50%B over 50 min (*ca.* 1%B/min) at a flow rate of 5 mL/min. Fractions were analysed by RP-HPLC and ESI-MS for compound identification. Fractions from multiple runs were combined and lyophilised to afford the desired compound, thanatin* (**5**), as a white amorphous powder (25.2 mg, 11% recovery [based on crude yield], > 95% purity, 6.9% overall yield).

ESI-MS: Mass calculated for [$C_{106}H_{176}N_{36}O_{26}S_2$] 2434.3; deconvoluted mass observed: 2435.3 ± 0.2. Charge states; 406.9 $[M+6H]^{6+}$, 488.1 $[M+5H]^{5+}$,609.8 $[M+4H]^{4+}$, 812.7 $[M+3H]^{3+}$.

HRMS (ESI/Q-TOF): mass calculated for $[C_{106}H_{176}N_{36}O_{26}S_2 + 4H]^{4+}$ 609.3307; mass observed: 609.3322.

RP-HPLC: t_R = 22.2 min. Phenomenex Luna C18 (100Å, 5 µm, 4.6 mm x 250 mm), linear gradient 5%B to 45%B over 40 min (*ca.* 1%B/min) at 1 mL/min.

Analogue **8**

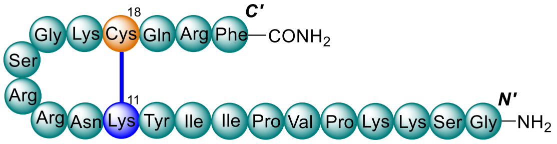

Following the functionalisation of the TentaGel®-S-NH_2 resin with Fmoc-Rink amide linker via **Method 1**, peptide **8** was synthesised by manual flow synthesis according to **Method 2** employing **Sub-Method 2.1** for the introduction of Cys(Trt) at position 18. Dde was orthogonally removed from Lys^{11} via **Method 3** and the allenamide handle was coupled via **Method 5** to the side chain of residue 11. The final linear peptide was liberated from the resin and protecting groups simultaneously removed following **Method 6**, to afford the crude linear peptide (260 mg, 70% yield [based on initial resin loading]). The crude linear peptide was cyclised via **Method 8** and quenched via **Method 9** to yield the crude desired peptide, **8**, at approximately 80% purity.

The crude peptide (132 mg), **8**, was dissolved in a solution of 1:9 MeCN/MQ H_2O at a concentration of ~10 mg/mL and purified (7 x ~2 mL injections) with a Phenomenex Luna C18 column (100 Å, 5 µm, 250 mm x 10 mm), employing a gradient with an isocratic phase of 5%B for 5 min, followed by a linear gradient to 50%B over 50 min (*ca.* 1%B/min) at a flow rate of 5 mL/min. Fractions were analysed by RP-HPLC and ESI-MS for compound identification. Fractions from multiple runs were combined and lyophilised to afford the desired compound, **8**, as a white amorphous powder (10.6 mg, 4.1% recovery [based on crude yield], > 98% purity, 2.8% overall yield).

ESI-MS: Mass calculated for [$C_{113}H_{187}N_{37}O_{27}S_1$] 2527.4; deconvoluted mass observed: 2528.1 ± 0.3. Charge states; 422.4 $[M+6H]^{6+}$, 506.6 $[M+5H]^{5+}$,633.0 $[M+4H]^{4+}$, 843.6 $[M+3H]^{3+}$.

RP-HPLC: t_R = 17.2 min. Phenomenex Luna C18 (100Å, 5 µm, 4.6 mm x 250 mm), linear gradient 5%B to 45%B over 40 min (*ca.* 1%B/min) at 1 mL/min.

Analogue **9**

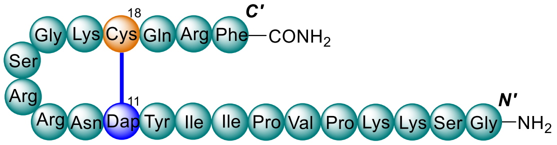

Following the functionalisation of the TentaGel®-S-NH_2 resin with Fmoc-Rink amide linker via **Method 1**, peptide **9** was synthesised by manual flow synthesis according to **Method 2** employing **Sub-Method 2.1** for the introduction of Cys(Trt) at position 18. N^{β}-Alloc was orthogonally removed from Dap^{11} via **Method 4** and the allenamide handle was coupled via **Method 5** to the side chain of residue 11. The final linear peptide was liberated from the resin and protecting groups simultaneously removed following **Method 6**, to afford the crude linear peptide (259 mg, 69% yield [based on initial resin loading]). The crude linear peptide was cyclised via **Method 8** and quenched via **Method 9** to yield the crude desired peptide, **9**, at approximately ~80% purity and 69% yield (based on initial resin loading).

The crude peptide (80 mg), **9**, was dissolved in a solution of 1:9 MeCN/MQ H_2O at a concentration of ~10 mg/mL and purified (4 x ~2 mL injections) with a Phenomenex Luna C18 column (100 Å, 5 µm, 250 mm x 10 mm), employing a gradient with an isocratic phase of 5%B for 5 min, followed by a linear gradient to 50%B over 50 min (*ca.* 1%B/min) at a flow rate of 5 mL/min. Fractions were analysed by RP-HPLC and ESI-MS for compound identification. Fractions from multiple runs were combined and lyophilised to afford the desired compound, **9**, as a white amorphous powder (5.1 mg, 2.0% recovery [based on crude yield], > 95% purity, 1.4% overall yield).

ESI-MS: Mass calculated for [$C_{110}H_{181}N_{37}O_{27}S_1$] 2485.4; deconvoluted mass observed: 2485.0 ± 0.3. Charge states; 498.2 $[M+5H]^{5+}$,622.4 $[M+4H]^{4+}$, 829.5 $[M+3H]^{3+}$.

RP-HPLC: t_R = 21.1 min. Phenomenex Luna C18 (100Å, 5 µm, 4.6 mm x 250 mm), linear gradient 5%B to 45%B over 40 min (*ca.* 1%B/min) at 1 mL/min.

Analogue **10**

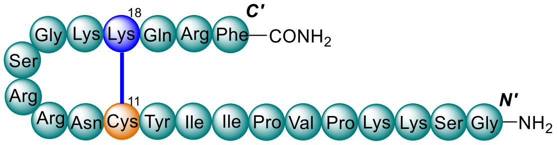

Following the functionalisation of the TentaGel®-S-NH_2 resin with Fmoc-Rink amide linker via **Method 1**, peptide **10** was synthesised by manual flow synthesis according to **Method 2** employing **Sub-Method 2.1** for the introduction of Cys(Trt) at position 11. Dde was orthogonally removed from Lys^{18} via **Method 3** and the allenamide handle was coupled via **Method 5** to the side chain of residue 18. The final linear peptide was liberated from the resin and protecting groups simultaneously removed following **Method 6**, to afford the crude linear peptide (175 mg, 47% [based on initial resin loading]). The crude linear peptide was cyclised via **Method 8** and quenched via **Method 9** to yield the crude desired peptide, **10**, at approximately > 80% purity.

The crude peptide (128 mg), **10**, was dissolved in a solution of 1:9 MeCN/MQ H_2O at a concentration of ~18 mg/mL and purified (4 x ~2 mL injections) with a Phenomenex Luna C18 column (100 Å, 5 µm, 250 mm x 10 mm), employing a gradient with an isocratic phase of 5%B for 5 min, followed by a linear gradient to 50%B over 50 min (*ca.* 1%B/min) at a flow rate of 5 mL/min. Fractions were analysed by RP-HPLC and ESI-MS for compound identification. Fractions from multiple runs were combined and lyophilised to afford the desired compound, **10**, as a white amorphous powder (20.8 mg, 12% recovery [based on crude yield], > 95% purity, 5.6% overall yield).

ESI-MS: Mass calculated for [$C_{113}H_{187}N_{37}O_{27}S_1$] 2527.4; deconvoluted mass observed: 2527.7 ± 0.2. Charge states; 506.6 $[M+5H]^{5+}$,632.9 $[M+4H]^{4+}$, 843.5 $[M+3H]^{3+}$.

RP-HPLC: t_R = 18.0 min. Phenomenex Luna C18 (100Å, 5 µm, 4.6 mm x 250 mm), linear gradient 5%B to 45%B over 40 min (*ca.* 1%B/min) at 1 mL/min.

Analogue **11**

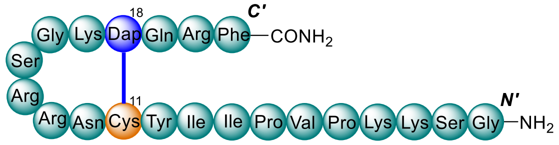

Following the functionalisation of the TentaGel®-S-NH_2 resin with Fmoc-Rink amide linker via **Method 1**, peptide **11** was synthesised by manual flow synthesis according to **Method 2** employing **Sub-Method 2.1** for the introduction of Cys(Trt) at position 11. N^{β}-Alloc was orthogonally removed from Dap^{18} via **Method 4** and the allenamide handle was coupled via **Method 5** to the side chain of residue 18. The final linear peptide was liberated from the resin and protecting groups simultaneously removed following **Method 6**, to afford 209 mg of the crude linear peptide (209 mg, 56% yield [based on initial resin loading]). The crude linear peptide was cyclised via **Method 8** and quenched via **Method 9** to yield the crude desired peptide, **11**, at approximately > 85% purity.

The crude peptide (135 mg), **11**, was dissolved in a solution of 1:9 MeCN/MQ H_2O at a concentration of ~8 mg/mL and purified (9 x ~2 mL injections) with a Phenomenex Luna C18 column (100 Å, 5 μm, 250 mm x 10 mm), employing a gradient with an isocratic phase of 5%B for 5 min, followed by a linear gradient to 50%B over 50 min (*ca.* 1%B/min) at a flow rate of 5 mL/min. Fractions were analysed by RP-HPLC and ESI-MS for compound identification. Fractions from multiple runs were combined and lyophilised to afford the desired compound, **11**, as a white amorphous powder (8.4 mg, 4.0% recovery [based on crude yield], > 98% purity, 3.4% overall yield).

ESI-MS: Mass calculated for [$C_{110}H_{181}N_{37}O_{27}S_1$] 2485.4; deconvoluted mass observed: 2486.1 ± 0.4. Charge states; 498.3 $[M+5H]^{5+}$,622.5 $[M+4H]^{4+}$, 829.6 $[M+3H]^{3+}$.

RP-HPLC: t_R = 20.6 min. Phenomenex Luna C18 (100Å, 5 μm, 4.6 mm x 250 mm), linear gradient 5%B to 45%B over 40 min (*ca.* 1%B/min) at 1 mL/min.

3.1 Spectral Data (HPLC/MS) of Purified Peptides 5, 8 – 11

3.1.1 HPLC Data for Analogues 5, 8 – 11

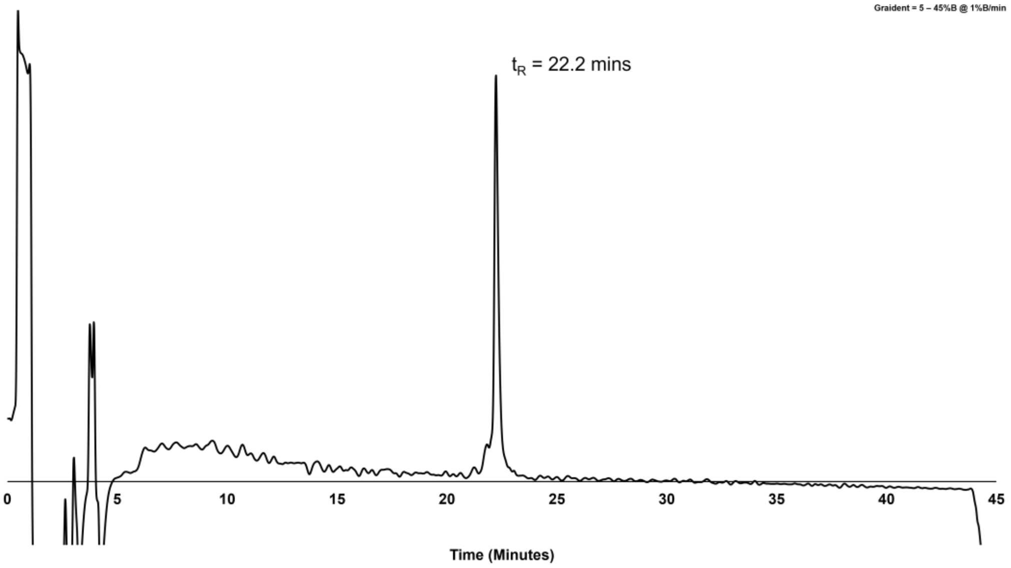

Figure S1. Analytical RP-HPLC chromatogram (214 nm) of purified peptide, thanatin* (**5**) (*ca.* 95% as analysed by peak area). Phenomenex Luna C18 (100Å, 5 µm, 4.6 mm x 250 mm), linear gradient 5%B to 45%B over 40 min (*ca.* 1%B/min) at 1 mL/min. t_R = 22.2 min.

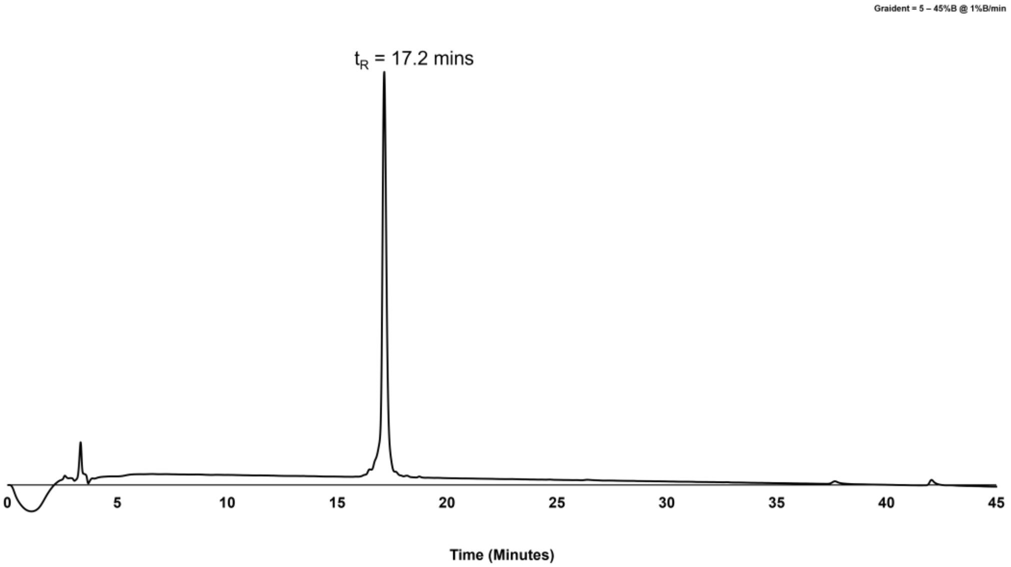

Figure S2. Analytical RP-HPLC chromatogram (214 nm) of purified peptide, **8** (*ca.* > 98% as analysed by peak area). Phenomenex Luna C18 (100Å, 5 µm, 4.6 mm x 250 mm), linear gradient 5%B to 45%B over 40 min (*ca.* 1%B/min) at 1 mL/min. t_R = 17.2 min.

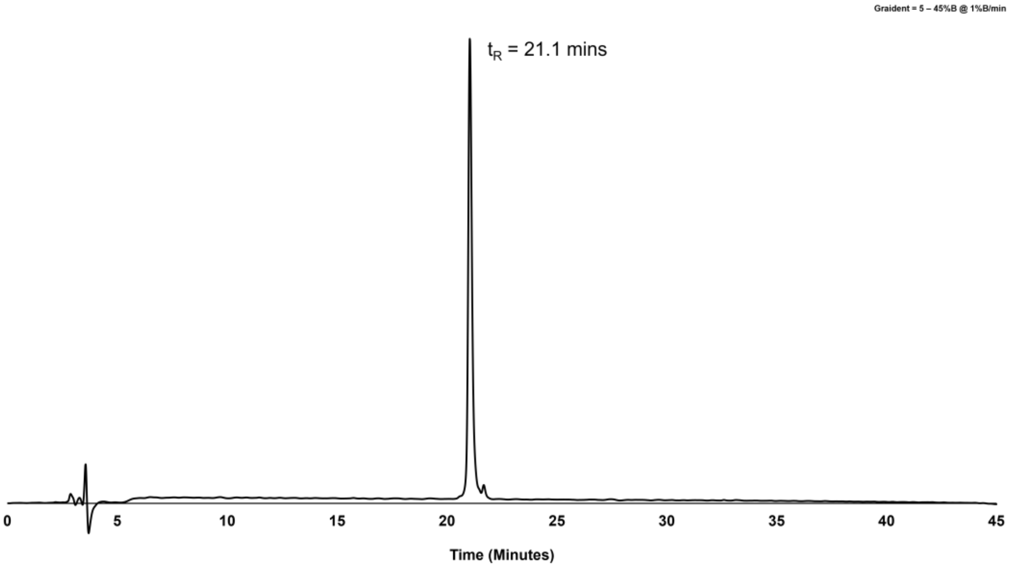

Figure S3. Analytical RP-HPLC chromatogram (214 nm) of purified peptide, **9** (*ca.* > 95% as analysed by peak area). Phenomenex Luna C18 (100Å, 5 µm, 4.6 mm x 250 mm), linear gradient 5%B to 45%B over 40 min (*ca.* 1%B/min) at 1 mL/min. t_R = 21.1 min.

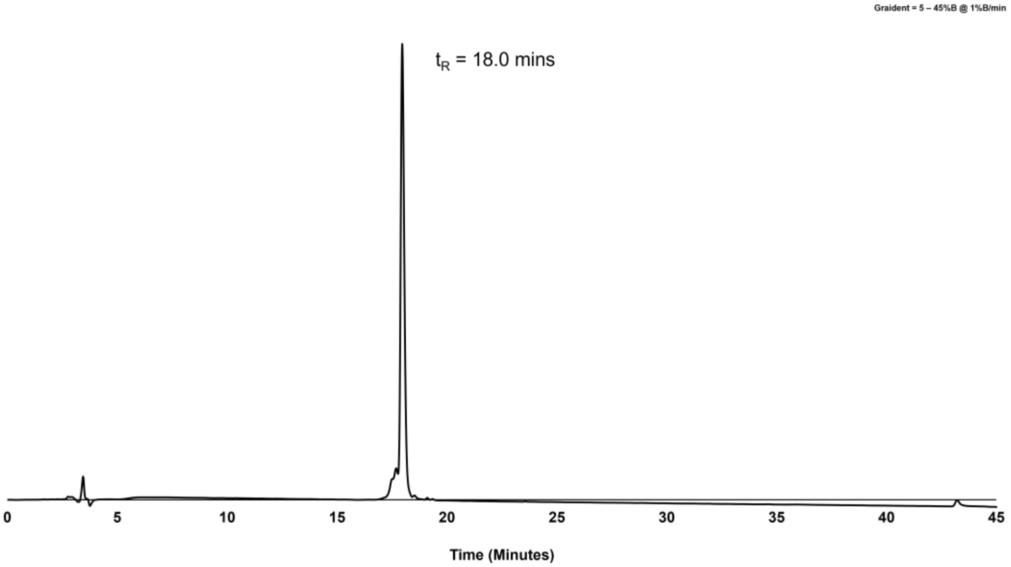

Figure S4. Analytical RP-HPLC chromatogram (214 nm) of purified peptide, **10** (*ca.* > 95% as analysed by peak area). Phenomenex Luna C18 (100Å, 5 µm, 4.6 mm x 250 mm), linear gradient 5%B to 45%B over 40 min (*ca.* 1%B/min) at 1 mL/min. t_R = 18.0 min.

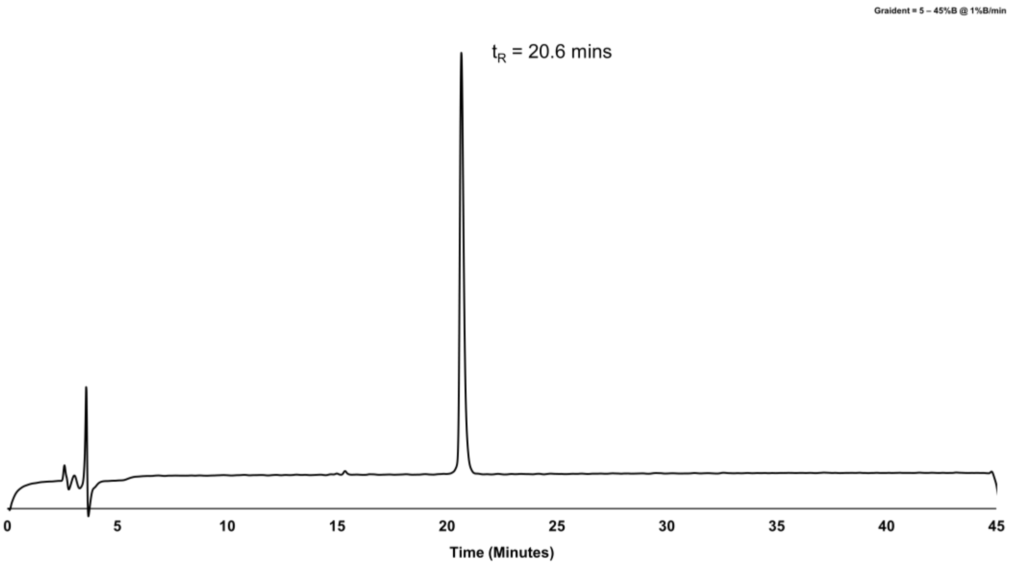

Figure S5. Analytical RP-HPLC chromatogram (214 nm) of purified peptide, **11** (*ca.* > 98% as analysed by peak area). Phenomenex Luna C18 (100Å, 5 µm, 4.6 mm x 250 mm), linear gradient 5%B to 45%B over 40 min (*ca.* 1%B/min) at 1 mL/min. t_R = 20.6 min.

3.1.2 ESI-MS Data for Analogues 5, 8 – 11

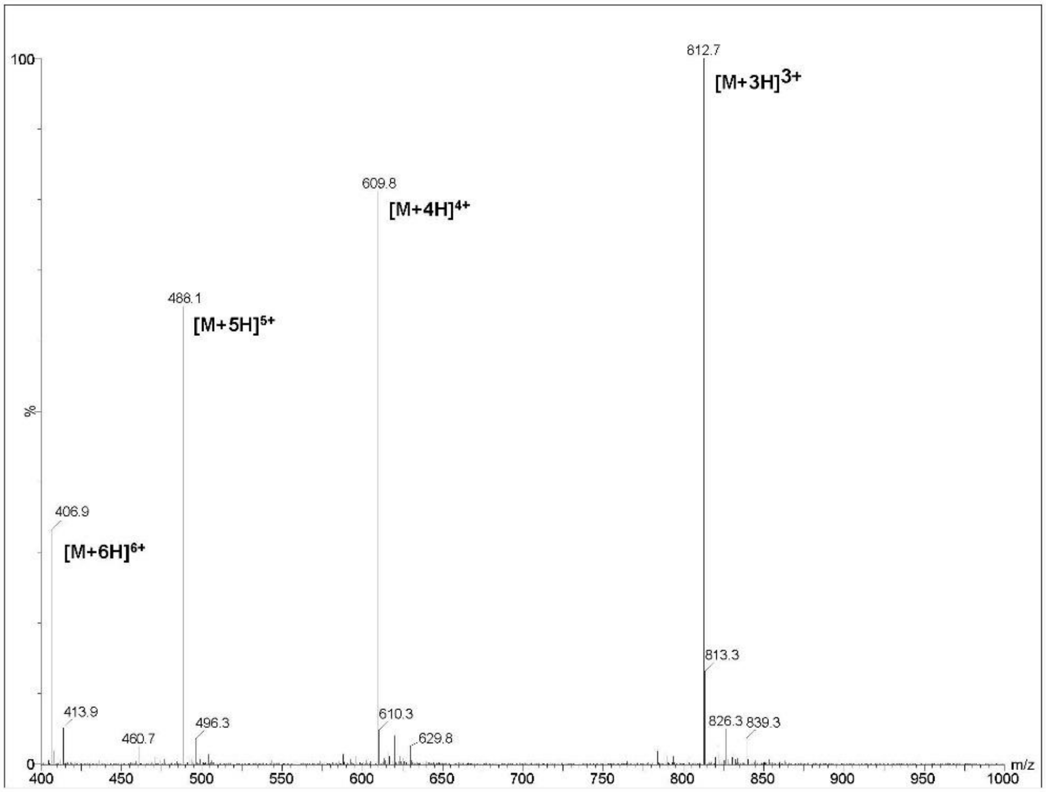

Figure S6. ESI-MS of purified peptide, thanatin*, **5**, mass calculated for [$C_{106}H_{176}N_{36}O_{26}S_2$] 2434.3; deconvoluted mass observed: 2435.3 ± 0.2. Charge states; 406.9 $[M+6H]^{6+}$, 488.1 $[M+5H]^{5+}$,609.8 $[M+4H]^{4+}$, 812.7 $[M+3H]^{3+}$.

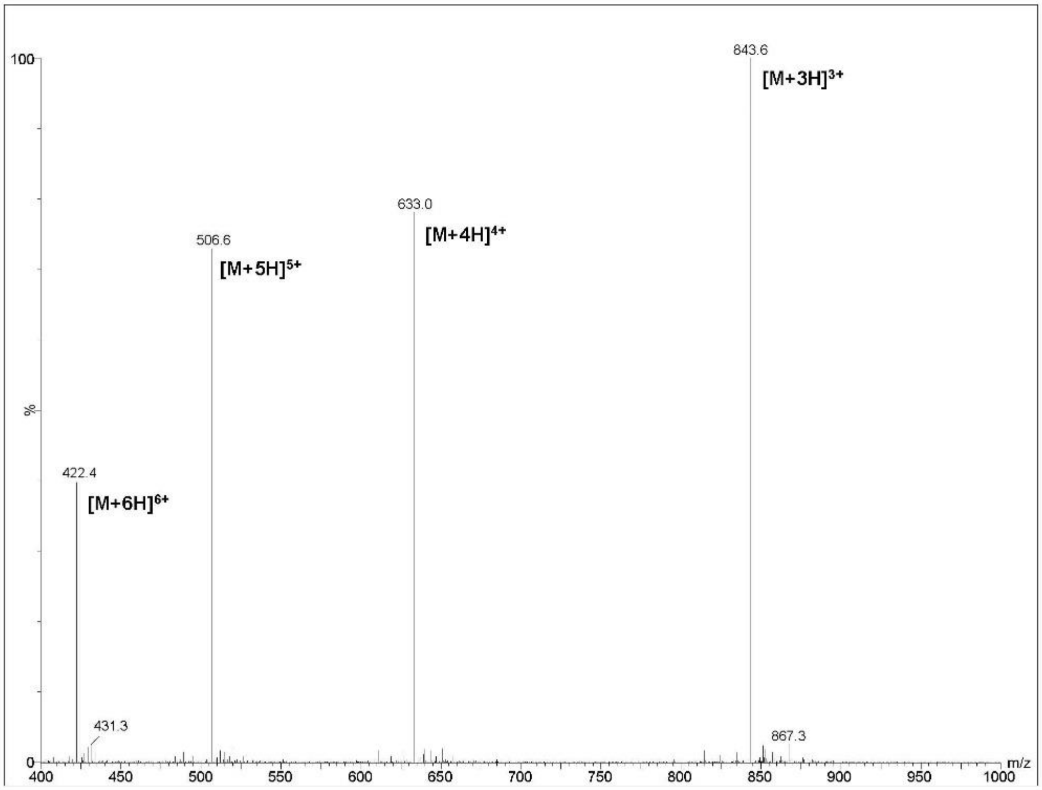

Figure S7. ESI-MS of purified peptide, **8**, mass calculated for [$C_{113}H_{187}N_{37}O_{27}S_1$] 2527.4; deconvoluted mass observed: 2528.1 ± 0.3. Charge states; 422.4 $[M+6H]^{6+}$, 506.6 $[M+5H]^{5+}$,633.0 $[M+4H]^{4+}$, 843.6 $[M+3H]^{3+}$.

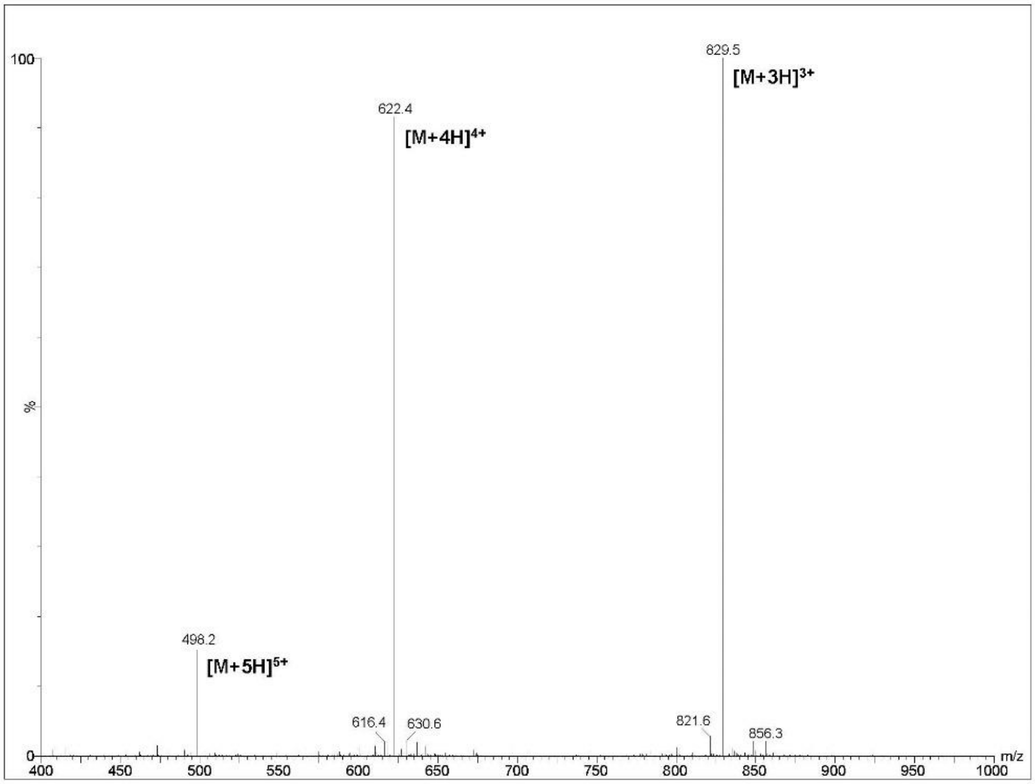

Figure S8. ESI-MS of purified peptide, **9**, mass calculated for [$C_{110}H_{181}N_{37}O_{27}S_1$] 2485.4; deconvoluted mass observed: 2485 ± 0.3. Charge states; 498.2 $[M+5H]^{5+}$,622.4 $[M+4H]^{4+}$, 829.5 $[M+3H]^{3+}$.

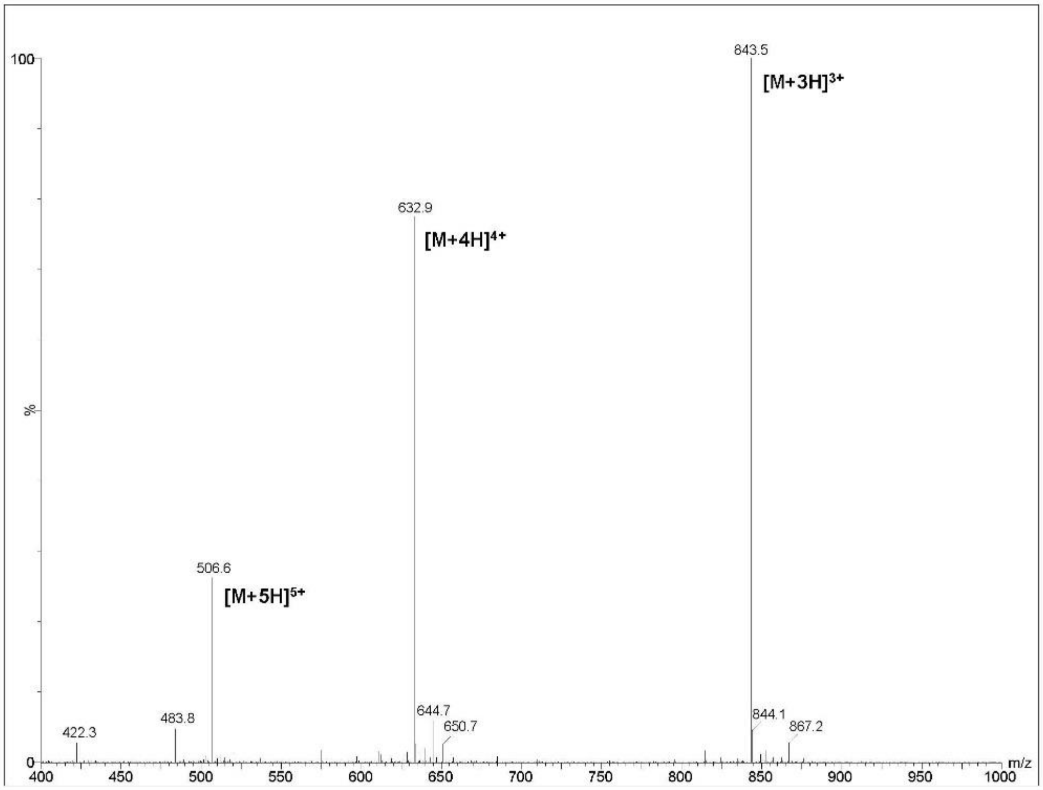

Figure S9. ESI-MS of purified peptide, **10**, mass calculated for [$C_{113}H_{187}N_{37}O_{27}S_1$] 2527.4; deconvoluted mass observed: 2527.7 ± 0.2. Charge states; 506.6 $[M+5H]^{5+}$,632.9 $[M+4H]^{4+}$, 843.5 $[M+3H]^{3+}$.

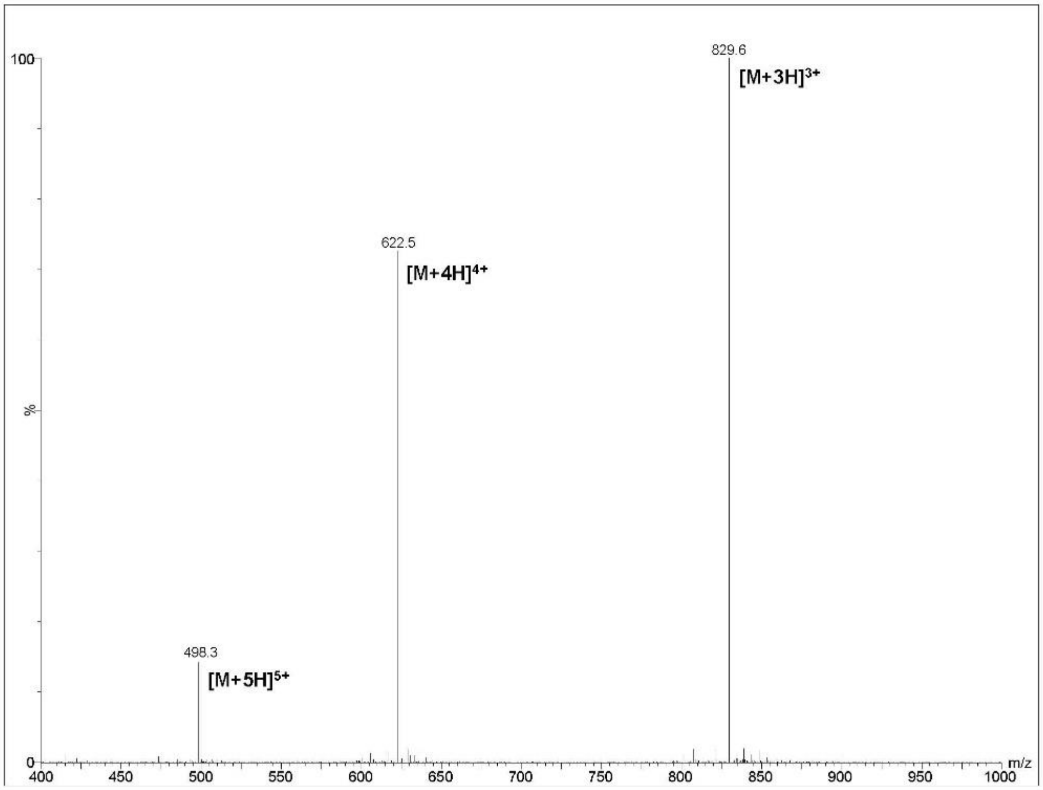

Figure S10. ESI-MS of purified peptide, **11**, mass calculated for [$C_{110}H_{181}N_{37}O_{27}S_1$] 2485.4; deconvoluted mass observed: 2486.1 ± 0.4. Charge states; 498.3 $[M+5H]^{5+}$,622.5 $[M+4H]^{4+}$, 829.6 $[M+3H]^{3+}$.

3.1.3 HRMS Data for Thanatin* (5)

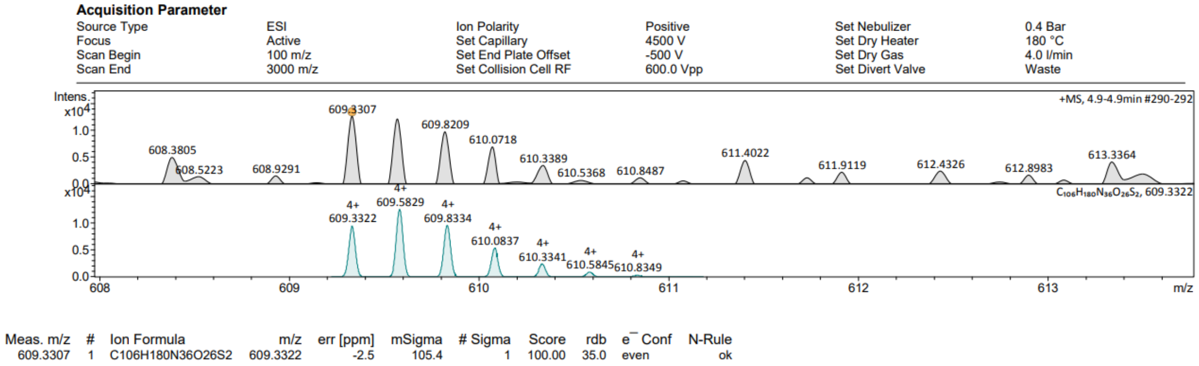

Figure S11. HRMS (ESI/Q-TOF) of purified thanatin* (**5**), formula analysis for [$C_{106}H_{176}N_{36}O_{26}S_2$ + 4H]$^{4+}$ 609.3307; observed: 609.3322.

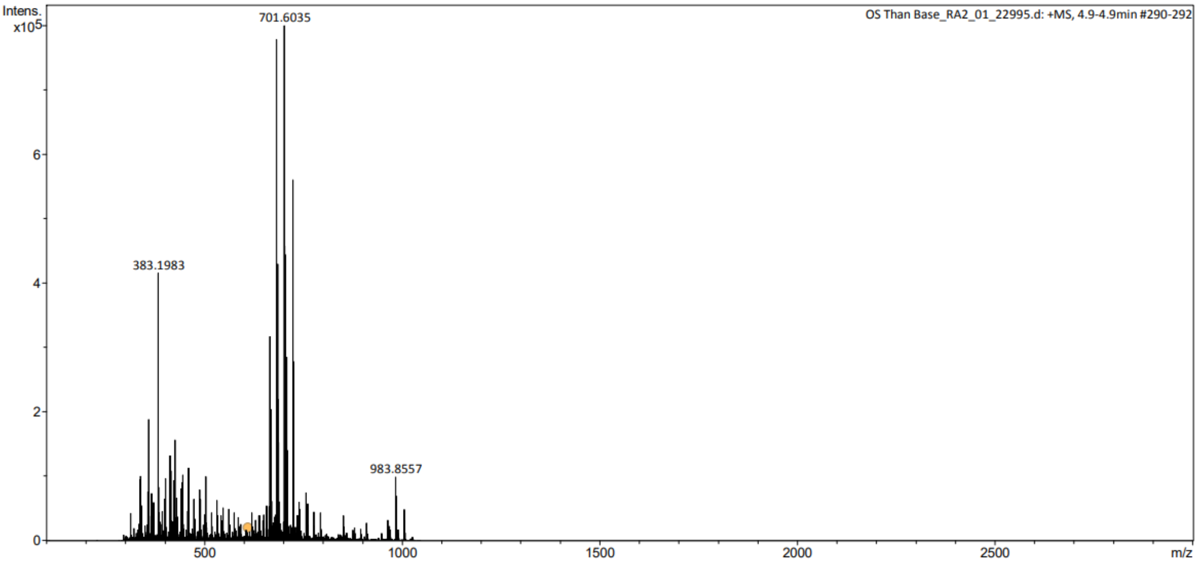

Figure S12. HRMS (ESI/Q-TOF), mass calculated for purified thanatin* (**5**), [$C_{106}H_{176}N_{36}O_{26}S_2$]: 2,433.3228; deconvoluted mass observed: 2433.3288.

4 Antimicrobial Testing of Thanatin* (5) and Analogues 8 – 11

4.1 Antibacterial Testing

Staphylococcus aureus ATCC 29213, *Pseudomonas aeruginosa* (SVB-B9) (type strain) and *Escherichia coli* ATCC 25922 were grown in cation adjusted Mueller Hinton (CAMHB) broth at 37 °C with shaking (200 rpm). MIC assays were performed in accordance with the CLSI recommended protocol (Clinical and Laboratory Standards Institute, 2006, Performance standards for antimicrobial susceptibility testing; sixteenth informational supplement. CLSI document M100-S16, Wayne, PA., 2006). Briefly**,** a two-fold dilution series of the test compounds (from 64 mM to 0.25 mM, final) was prepared in triplicate in polypropylene 96-well plates in CAMHB. Fresh cultures of bacteria grown for *ca. 6* h were diluted accordingly in fresh media before adding 50 µL of inoculum to each well of the MIC plate, to achieve a final volume of 100 µL with a uniform CFU/ml of ~5 x10^5 in each well. A growth control (untreated) and sterility control (non-inoculated) well was included for each test compound replicate. Plates were incubated at 37 °C with shaking for 18 h before determining the MIC. MIC values were determined as the lowest concentration at which no growth was observed consistently across all three biological replicates of the assay and within triplicates of each test compound.

4.2 Antifungal Testing

Antimicrobial susceptibility of *Candida albicans* SC5314 (type strain) was assessed by broth microdilution in accordance with the CLSI recommended protocols (Clinical and Laboratory Standards Institute, 2006, Performance standards for antimicrobial susceptibility testing; sixteenth informational supplement. CLSI document M100-S16, Wayne, PA., 2006). Briefly, a two-fold dilution series of the test compounds (from 64 µM to 0.25 µM, final) was prepared in triplicate in polypropylene 96-well plates, using RPMI 1640 media (with glutamine and phenol red, without bicarbonate). Inoculum was prepared from a 24 h old culture by picking 5 colonies (~1 mm in diameter) and resuspending these in 0.85% saline. Upon vortex mixing, the resulting suspension was diluted to provide a solution with absorbance equivalent to that of a 0.5 McFarland standard, approx. 0.1 OD at 530 nm (using 1 cm path length cuvette). This suspension was then diluted 1:50 and a further 1:20 with RPMI 1640 media to as to achieve a final inoculum of 0.5 x 10^3 to 2.5 x 10^3 CFU/mL upon addition to the plate. The plates were incubated at 35 °C for 48 h, at which time the MIC was determined as the lowest test concentration at which no turbidity was observed across all three replicates. The assay was performed independently on three occasions and the reported MIC defined as the lowest concentration in which agreement was observed for all three biological replicates.

Synergism with Polymyxin B

Synergistic activities between combinations of AMPs were examined for *Escherichia coli* ATCC 25922. Synergy was determined by measuring the fractional inhibitory concentration index (FICI) using a checkerboard synergy assay (Odds, 2003). Stocks of antibacterial agents were prepared at 4-fold the desired initial concentrations, prior to being serial dilute 2-fold, twice, first horizontally and then vertically. Bacterial strains were grown in cation adjusted Mueller Hinton (CAMHB) broth at 37 °C with shaking (200 rpm). Cultures of bacteria grown for *ca.* 6 h were diluted accordingly in fresh media before adding 50 µL of inoculum to each well of the MIC plate, to achieve a final volume of 100 µL with a uniform CFU/ml of ~5 x10^5 in each well. Plates were incubated at 37 °C with shaking for 18 h before determining the MIC. MIC values were determined as the lowest concentration at which no growth was observed. The equations used for determining FICI was as follows.

$$FIC\ Index = FIC\ A + FIC\ B\ = \frac{MIC_A^{Combination}}{MIC_A^{Alone}} + \frac{MIC_B^{Combination}}{MIC_B^{Alone}}$$

The MIC was determined as the wells without any visible growth. Synergy was defined as occurring at an FIC ≤ 0.5 (teal), antagonism at FIC > 4.0 and an additive effect in the range 0.5 – 4.0 (yellow) (Odds, 2003).

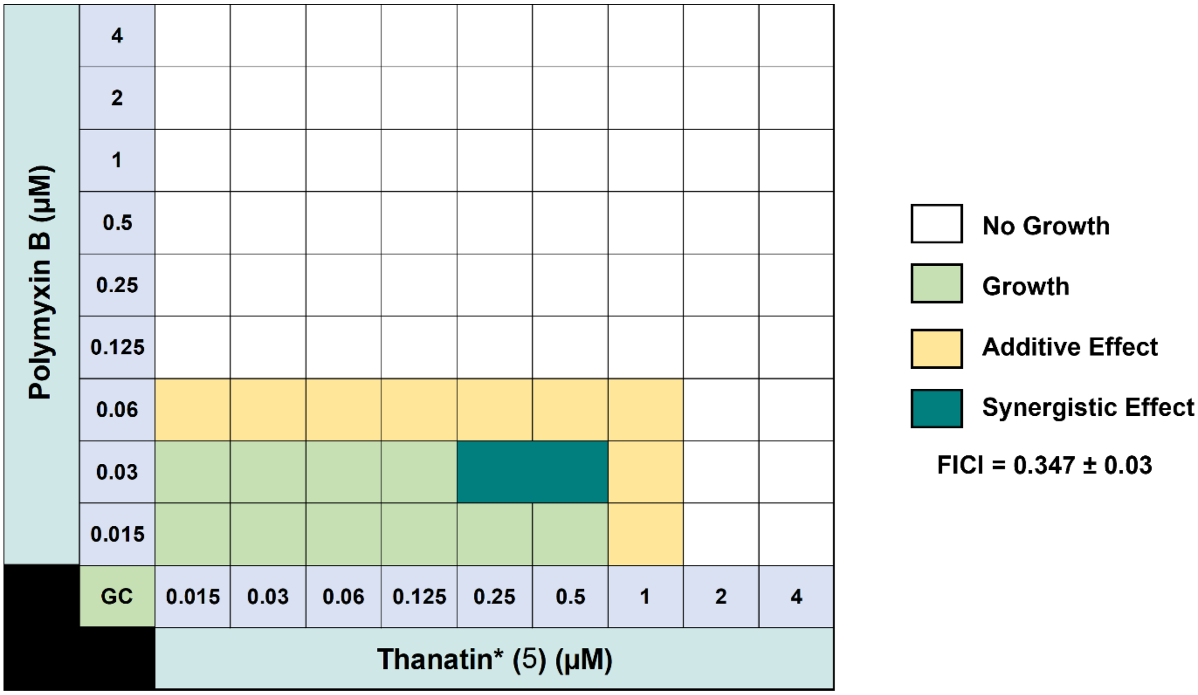

Figure S13. Example of one of three synergism plots for thanatin* (**5**) and polymyxin B against *E. coli*. FICI calculated across three repeats to be an average of 0.347 with a standard deviation of 0.03, indicating synergism occurring.

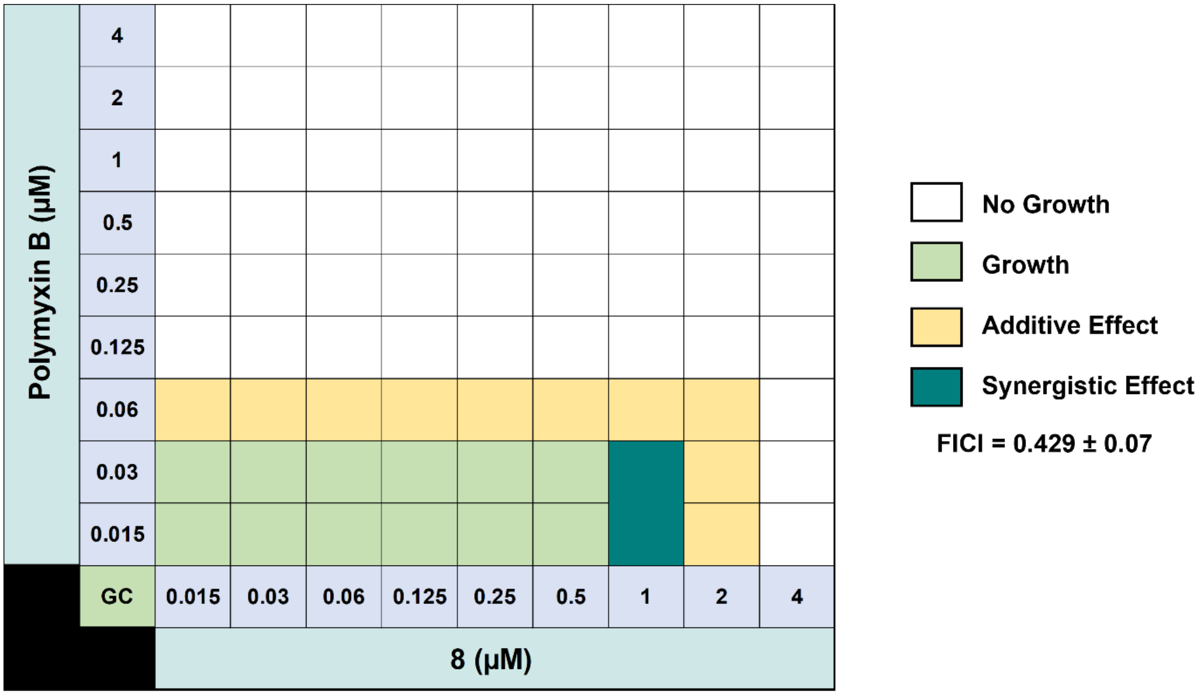

Figure S14. Example of one of three synergism plots for analogue **8** and polymyxin B against *E. coli*. FICI calculated across three repeats to be an average of 0.429 with a standard deviation of 0.07, indicating synergism occurring.

5 Circular Dichroism

All CD spectra were recorded using a Chirascan CD spectrometer (Applied Photophysics Ltd., Leatherhead, UK) at 20 °C with a cuvette of 1 mm path length (106-QS, Hellma Analytics, Mullheim, Germany) in the range from 180 and 260 nm at 0.5 nm intervals with a time-to-point of 0.5 s. Each peptide sample was prepared to a concentration of 50 µM in the respective solvents. Each spectrum was prepared from an average of five scans obtained with a 1 nm optical bandwidth. The baseline scans were collected with the solvent alone, averaged, and then subtracted from the sample scans. Raw data was exported to excel for processing and expressed as mean residue molar ellipticities [θ] in (deg.cm^2/dmol) and calculated as $\theta = S / (10 \times c \times L \times n)$, where S is the raw CD signal in millidegrees, c is the peptide concentration (M), L is the cuvette path length (cm), and n is the number of peptide bonds.

6 Supplementary Figures and Tables

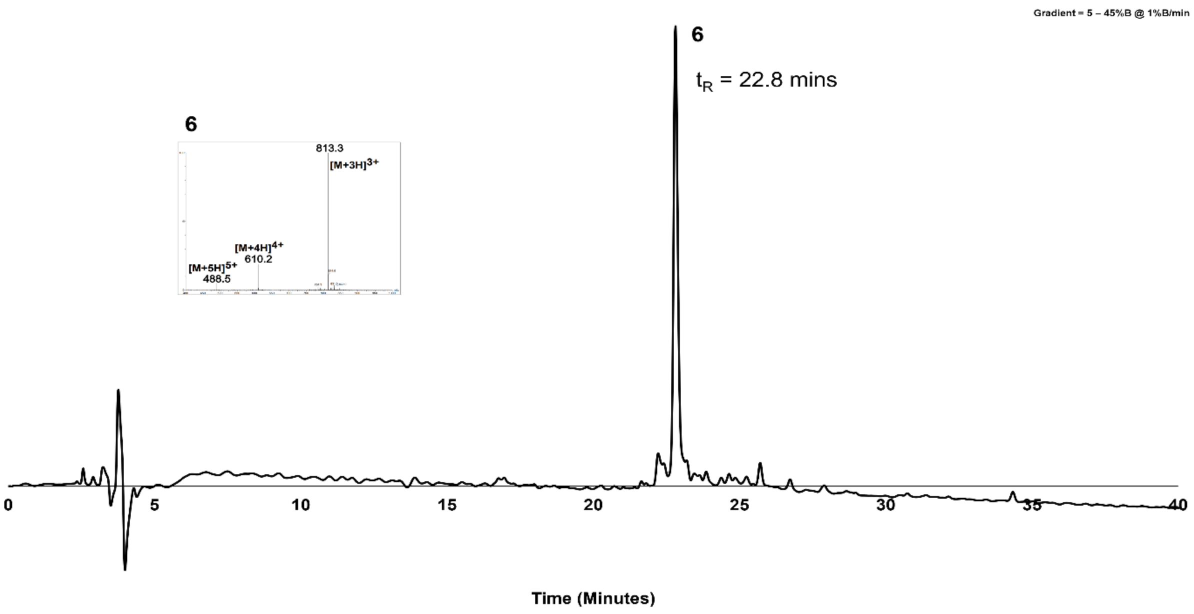

Figure S15. Analytical RP-HPLC chromatogram (214 nm) of linear peptide **6** prepared by fast flow Fmoc-SPPS. Phenomenex Luna C18 (100Å, 5 µm, 4.6 mm x 250 mm), linear gradient 5%B to 45%B over 40 min (*ca.* 1%B/min) at 1 mL/min. ESI-MS inset.

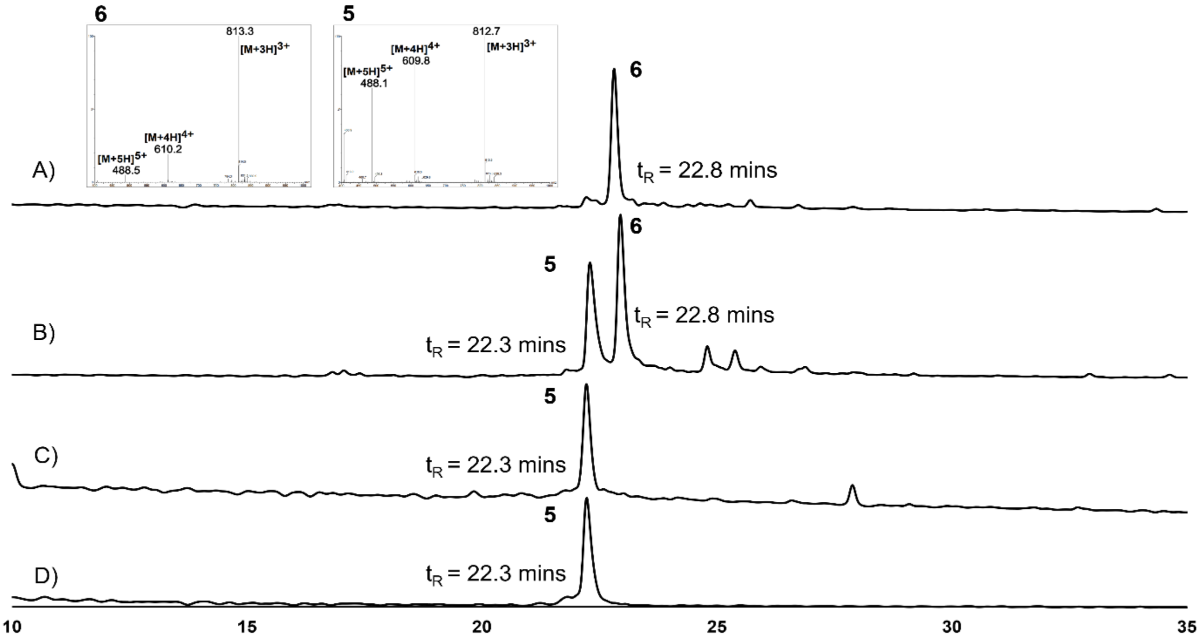

Figure S16. Analytical RP-HPLC chromatograms (214 nm) of thanatin* (**5**) oxidation by NCS. A) Linear peptide **6** prior to treatment with NCS, B) post NCS addition (t = 15 mins), C) post NCS addition (t = 60 mins) and D) purified peptide **5**. Phenomenex Luna C18 (100Å, 5 µm, 4.6 mm x 250 mm), linear gradient 5%B to 45%B over 40 min (*ca.* 1%B/min) at 1 mL/min. ESI-MS inset (left = **6,** right = **5).**

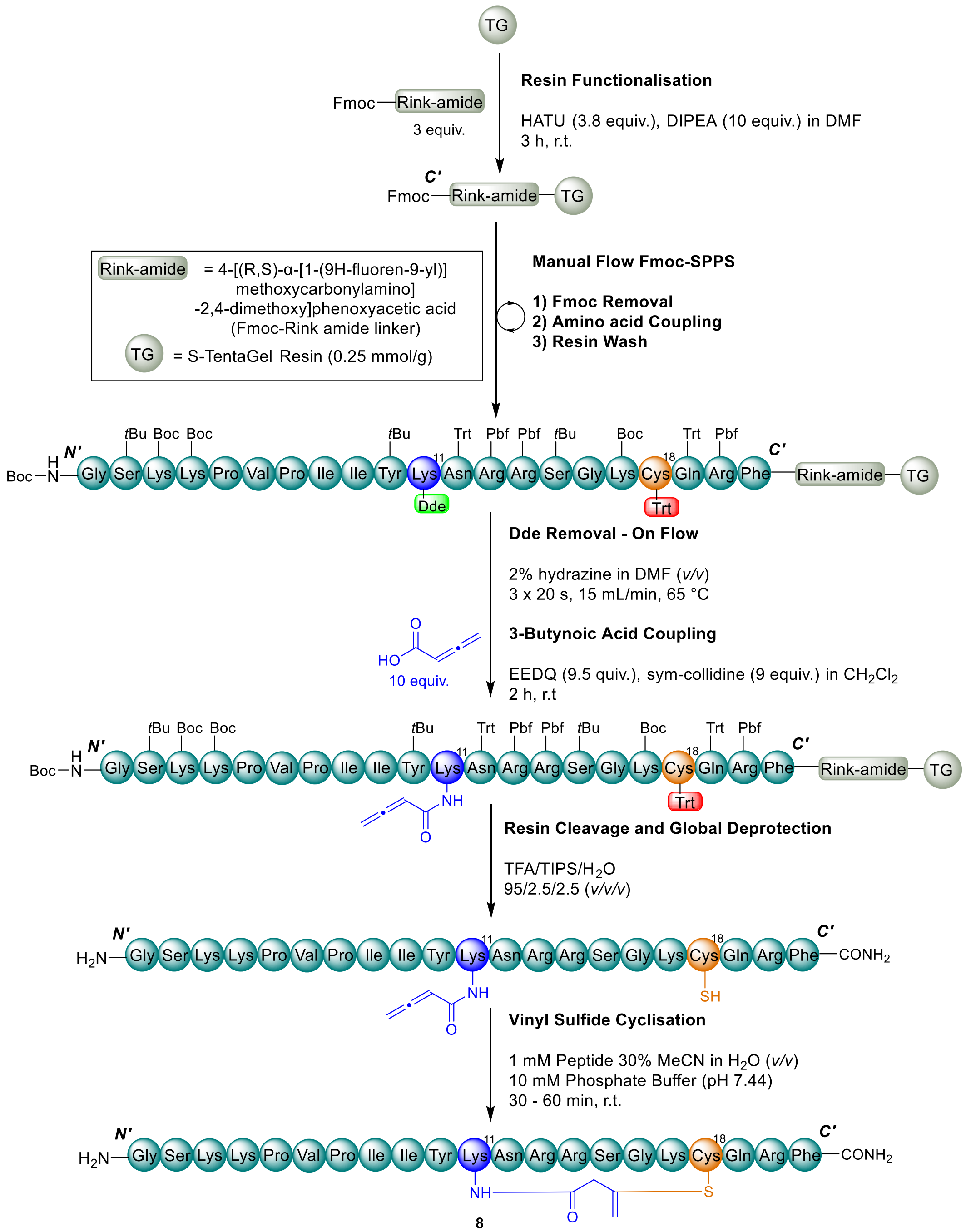

Scheme S1. Synthesis of analogue **8** presented as a representative of the synthetic approach for Cys substitution with Lys(Dde). Cys residues and thiols (orange), Cys(Trt) protection (red), Dde protection (bright green) and substituted Cys residue, allenamidyl handle and vinyl sulfide bridge (blue). (1) 40 s flow (15 mL/min) 30% (v/v) piperidine in DMF; (2) AA coupling following protocol 2A for all non Cys AAs and protocol 2B for Cys residues; 2A) 30 s flow of AA coupling solution (0.3 M AA, 0.28 M HATU, 20 equiv. DIPEA) at 15 mL/min.; 2B) 40 s flow of Cys(Trt) (0.3 M AA 0.28 M PyAOP, 20 equiv. sym-collidine) at 15 mL/min. (3) 40 s flow of DMF at 15 mL/min. All flow reactions were performed at 65 °C.

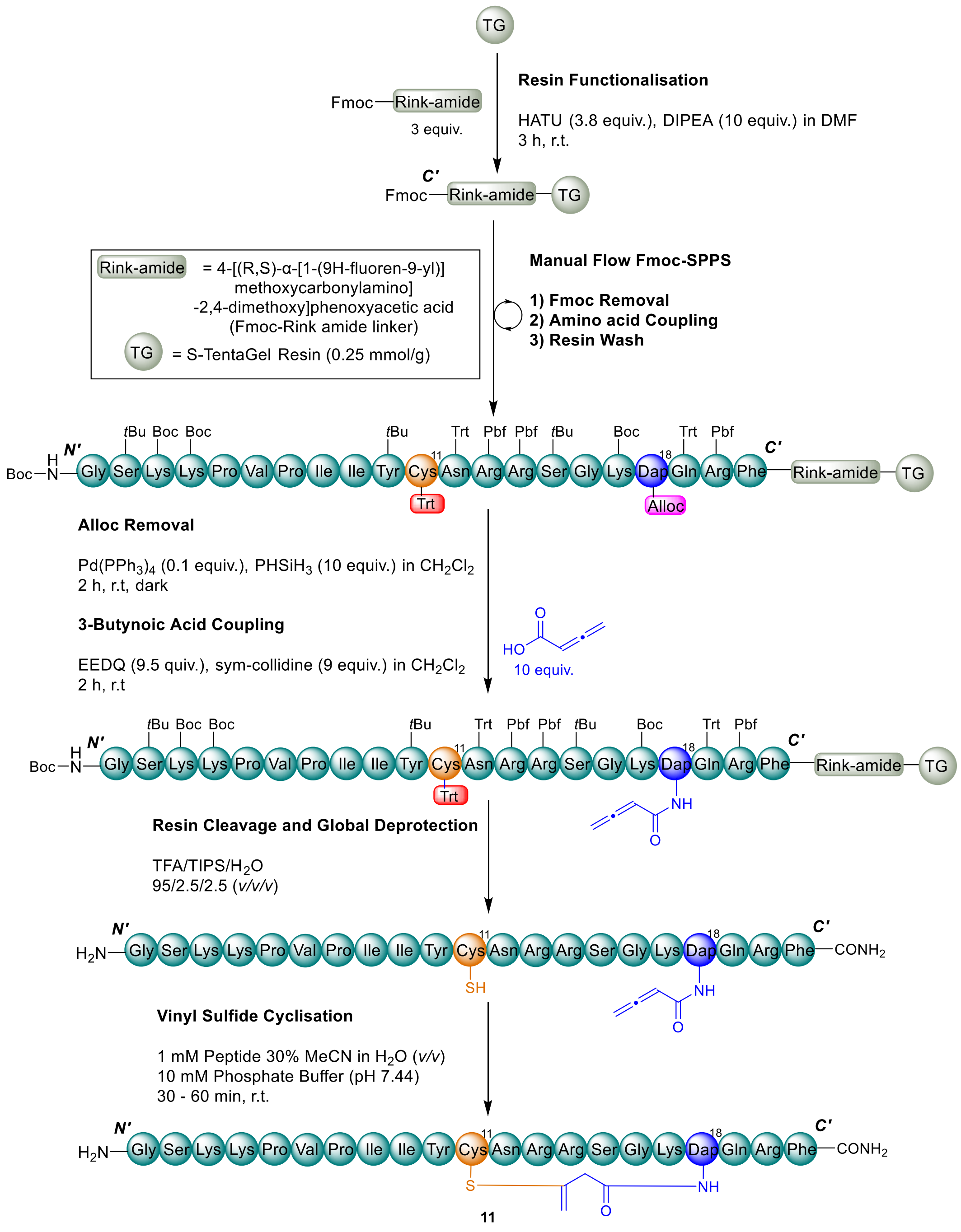

Scheme S2. Synthesis of analogue **11** presented as a representative of the synthetic approach for Cys substitution with Dap(Alloc). Cys residues and thiols (orange), Cys(Trt) protection (red), N^{β}-Alloc protection (pink) and substituted Cys residue, allenamidyl handle and vinyl sulfide bridge (blue). (1) 40 s flow (15 mL/min) 30% (v/v) piperidine in DMF; (2) AA coupling following protocol 2A for all non Cys AAs and protocol 2B for Cys residues; 2A) 30 s flow of AA coupling solution (0.3 M AA, 0.28 M HATU, 20 equiv. DIPEA) at 15 mL/min.; 2B) 40 s flow of Cys(Trt) (0.3 M AA 0.28 M PyAOP, 20 equiv. sym-collidine) at 15 mL/min. (3) 40 s flow of DMF at 15 mL/min. All flow reactions were performed at 65 °C.

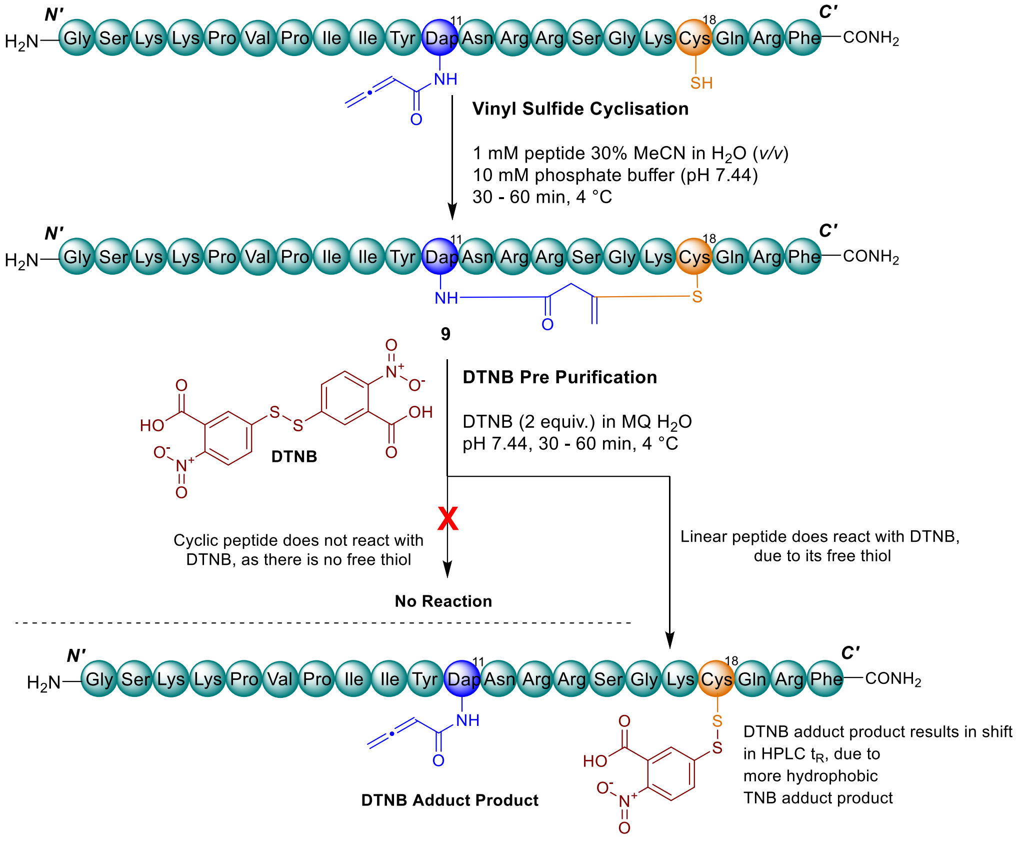

Scheme S3. Cyclisation to form analogue **9**, with addition of DTNB pre-purification. Cys residues and thiols (orange), TNB adduct (brown) and substituted Cys residue, allenamidyl handle and vinyl sulfide bridge (blue).

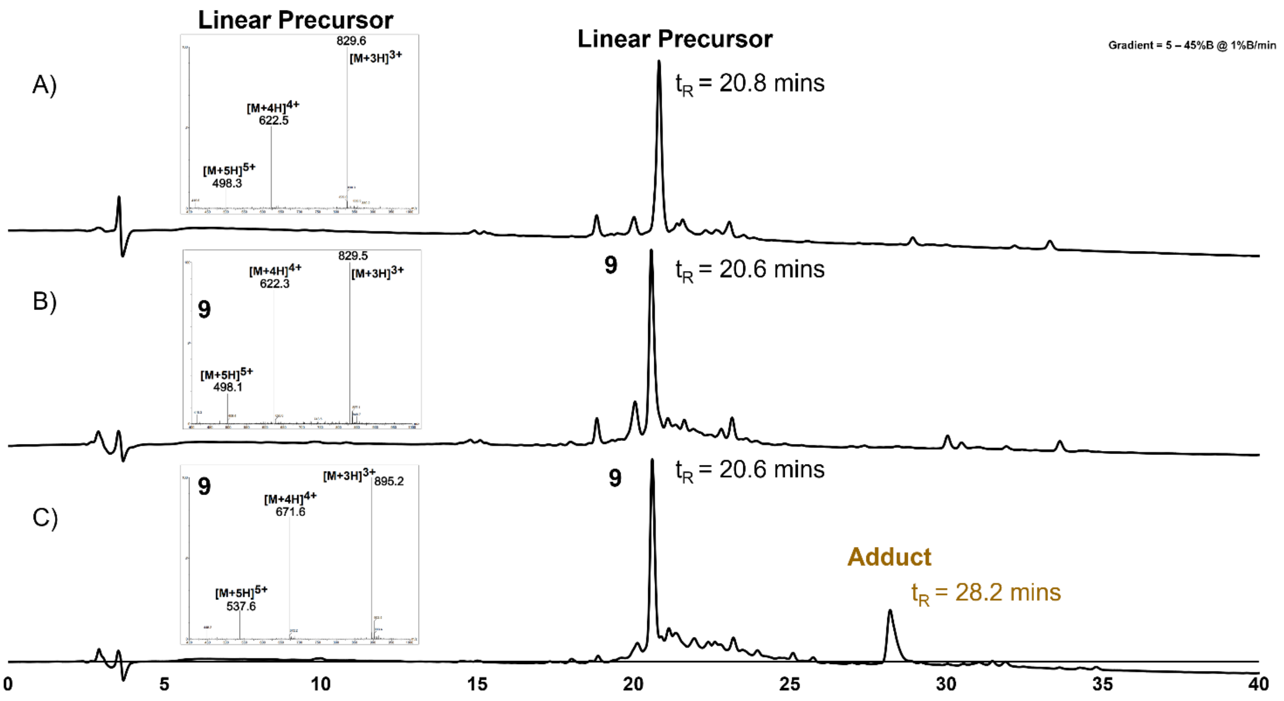

Figure S17. Analytical RP-HPLC chromatograms (214 nm) of analogue **9**. A) Post cleavage and global deprotection, B) post cyclisation, and C) post cyclisation + DTNB. Phenomenex Luna C18 (100Å, 5 µm, 4.6 mm x 250 mm), linear gradient 5%B to 45%B over 40 min (*ca.* 1%B/min) at 1 mL/min.

Table S1. Summary of thanatin peptides synthesised herein.

Peptide (#)	t_R (min)[a]	*m/z* calculated	*m/z* observed (deconvoluted)	Purity (> %)	% Yield
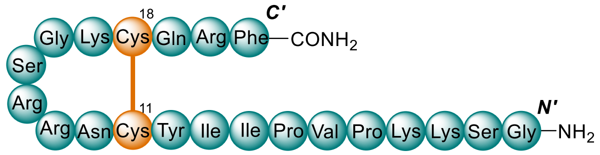 **5**	22.2	2434.3	2435.3 ± 0.2	95	6.9
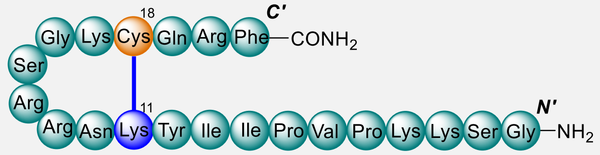 **8**	17.2	2527.4	2528.1 ± 0.3	98	2.8
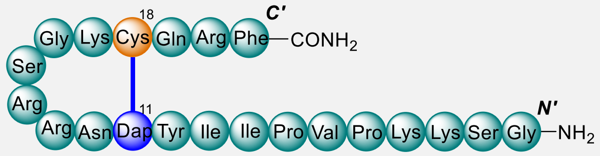 **9**	21.1	2485.4	2485.0 ± 0.3	95	1.4
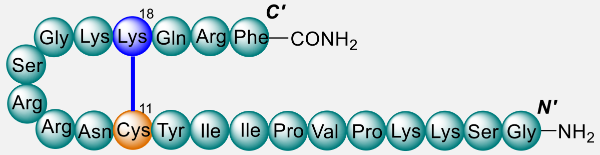 **10**	18.0	2527.4	2527.7 ± 0.2	95	5.6
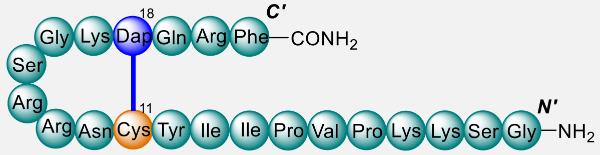 **11**	20.6	2485.4	2486.1 ± 0.4	98	3.4

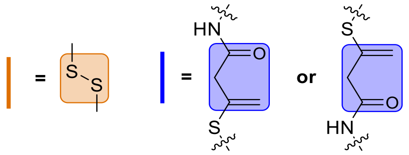

Phenomenex Luna C18 (100Å, 5 µm, 4.6 mm x 250 mm), linear gradient 5%B to 45%B over 40 min (*ca.* 1%B/min) at 1 mL/min.

7 References

Abdel Monaim, S. A. H., Ramchuran, E. J., El-Faham, A., Albericio, F., and de la Torre, B. G. (2017). Converting Teixobactin into a Cationic Antimicrobial Peptide (AMP). *J. Med. Chem.* 60, 7476–7482. doi: 10.1021/acs.jmedchem.7b00834

Cameron, A. J., Harris, P. W. R., and Brimble, M. A. (2020). On-Resin Preparation of Allenamidyl Peptides: A Versatile Chemoselective Conjugation and Intramolecular Cyclisation Tool. *Angewandte Chemie* 132, 18210–18217. doi: 10.1002/ange.202004656

Clinical and Laboratory Standards Institute, 2006, Performance standards for antimicrobial susceptibility testing; sixteenth informational supplement. CLSI document M100-S16, Wayne, PA. (2006).

Odds, F. C. (2003). Synergy, antagonism, and what the chequerboard puts between them. *Journal of Antimicrobial Chemotherapy* 52, 1. doi: 10.1093/jac/dkg301

Postma, T. M., and Albericio, F. (2013). N-Chlorosuccinimide, an Efficient Reagent for On-Resin Disulfide Formation in Solid-Phase Peptide Synthesis. *Org. Lett.* 15, 616–619. doi: 10.1021/ol303428d
